# Systems Biology Approaches to Investigate Genetic and Epigenetic Molecular Progression Mechanisms for Identifying Gene Expression Signatures in Papillary Thyroid Cancer

**DOI:** 10.3390/ijms20102536

**Published:** 2019-05-23

**Authors:** Shan-Ju Yeh, Chien-Yu Lin, Cheng-Wei Li, Bor-Sen Chen

**Affiliations:** Laboratory of Automatic Control, Signal Processing and Systems Biology, Department of Electrical Engineering, National Tsing Hua University, Hsinchu 30013, Taiwan; m793281@gmail.com (S.-J.Y.); s105061632@m105.nthu.edu.tw (C.-Y.L.); cwlitw@gmail.com (C.-W.L.)

**Keywords:** papillary thyroid cancer, genetic and epigenetic network, next-generation sequencing (NGS) data, carcinogenic biomarkers, genetic and epigenetic drug targets, drug data mining

## Abstract

Thyroid cancer is the most common endocrine cancer. Particularly, papillary thyroid cancer (PTC) accounts for the highest proportion of thyroid cancer. Up to now, there are few researches discussing the pathogenesis and progression mechanisms of PTC from the viewpoint of systems biology approaches. In this study, first we constructed the candidate genetic and epigenetic network (GEN) consisting of candidate protein–protein interaction network (PPIN) and candidate gene regulatory network (GRN) by big database mining. Secondly, system identification and system order detection methods were applied to prune candidate GEN via next-generation sequencing (NGS) and DNA methylation profiles to obtain the real GEN. After that, we extracted core GENs from real GENs by the principal network projection (PNP) method. To investigate the pathogenic and progression mechanisms in each stage of PTC, core GEN was denoted in respect of KEGG pathways. Finally, by comparing two successive core signaling pathways of PTC, we not only shed light on the causes of PTC progression, but also identified essential biomarkers with specific gene expression signature. Moreover, based on the identified gene expression signature, we suggested potential candidate drugs to prevent the progression of PTC with querying Connectivity Map (CMap).

## 1. Introduction

Thyroid cancer is the most common endocrine malignancy and usually originates in follicular or parafollicular C cells. This is a disease in which cells grow abnormally and have the potential to spread to other parts of the body. Its symptoms can include swelling or a lump in the neck. In 2015, there were 3.2 million people living with thyroid cancer [1], resulting in 31,900 deaths [2]. It most commonly occurs on women whose ages are between 35 and 65 that are affected more often 3–4 times than men [3]. Besides, the incidence of thyroid cancer is growing faster than other cancers in the United States and other countries [4]. Papillary thyroid cancer (PTC) is a differentiated thyroid cancer (DTC) and accounts for approximately 85% of thyroid cancer forms [5]. It is usually discovered as an asymptomatic thyroid nodule on routine examination and is also discovered when detected as a hard nodule in a multinodular goiter or in enlarged cervical lymph nodes. Generally, thyroid cancer is related to the interaction between innate genes and acquired environmental factors. In addition, the suspected exposure to free radiation may cause mutations in thyroid cells or other etiological factors such as iodine-131 or thyroid inflammation, and other thyroid symptoms may also be converted to thyroid cancer [6]. At present, the main methods for the diagnosis of thyroid cancer are fine-needle aspiration biopsy (FNA), ultrasound imaging, and nuclear scan [6]. The most commonly used PTC therapy is thyroidectomy, followed by partial radioactive iodine residual ablation and thyroid hormone suppression therapy [5].

The tumor microenvironment is a complex milieu consisting of factors that promote growth and inhibit it, as well as nutrients, chemokines, and other noncancerous cell types [7]. The formation of cancer is mainly due to genetic and epigenetic regulations and the changes of microenvironment. Notably, doing post-transcriptional regulation on certain genes by miRNAs is one of epigenetic phenomena which may cause the carcinogenic progression of tumor cells. Moreover, microRNAs (miRNAs), a small noncoding RNAs containing ~21–23 nucleotides [8], can act as tumor suppressors or tumor promoters; critical factors in the development of cancer. In addition, genetic or epigenetic modification of miRNAs can drive them to have abnormal expressions in certain cancers and diseases, which play an important role in the cell dysfunctions including proliferation, differentiation, migration, and cell death [9].

Epigenetic modifications are important for the mechanisms of human gene expression and cell development. Inappropriate epigenetic modifications could trigger various signaling pathways to induce cellular dysfunctions resulting in cancer eventually [10]. For instance, abnormal DNA methylation would change the genome expression levels to induce cellular dysfunctions. Besides, a low DNA methylation may activate proto-oncogenes and cause the development of cancer cells. A high rate of DNA methylation may silence gene expression, which is also one of the keys to cancer progression. It has been reported that epigenetics could cause some gene expression changes in differentiated thyroid cancer [11,12]. In addition to DNA methylation, we also consider the effects of other epigenetic regulations of proteins in the signaling pathways such as phosphorylation, acetylation, deacetylation, methylation, ubiquitination, and mutation, which could be observed by the changes of basal level in the system model we constructed.

Along with the advances in high-throughput technologies, developing systematic methods to leverage genome-wide data efficiently is indispensable. The increasing use of systems biology approaches allows researchers to integrate heterogeneous data and understand disease from the system-level [13]. Common approaches using protein–protein interactions (PPIs) or gene ontologies drive the search for modules or terms that could function as gene signatures [14]. Based on systems biology, several mathematical techniques have been developed to analyze the systematic properties of complex biological networks [15]. Network-based classification by greedy search found subnetworks providing a novel hypothesis for pathways involved in tumor progression [16]. Integrating mRNA expression data and the human protein interaction analyses, quantitative framework was constructed to compare and contrast disease for identifying common disease-state signature [17].

Currently, drug combinations draw researchers’ attention because of biomarker discovery. The corresponding researches show higher efficacy, fewer side effects and less toxicity compared with single-drug treatments [18,19,20,21]. Moreover, previous studies have demonstrated that combination approaches improve safety and enhance clinical activity when dose, schedule, and configuration of each agent are properly evaluated, especially in combination immunotherapy [22]. Connectivity Map (CMap) build 02, a projected developed by the Broad Institute, contains 6100 instances with 1309 drugs and 156 concentrations on five cell lines. It has been widely used on drug discovery and drug repurposing/repositioning. Lim et al. found that histone deacetylase inhibitors (HDACs), including vorinostat and trichostatin A, were potential candidate drugs for treating gastric cancer by using a gastric cancer gene signature to query CMap [23]. A gene expression signature representing the biological effect of a combination of known drugs was analyzed by CMap to repurpose drugs for bipolar disorder [24]. In this study, we take advantage of CMap to find potential compounds for preventing the deterioration of PTC based on the identified biomarkers with specific gene expression signature.

The object of this study is to apply systems biology approaches including system modeling, reversed engineering techniques, system identification, system order detection, and principal network projection (PNP) methods on genome-wide next sequencing data for understating the genetic and epigenetic progression molecular mechanisms in PTC. Consequently, the identified abnormal gene expression signatures belonging to normal thyroid cells to early stage of PTC and early to late stage of PTC have been used to explore potential compounds, which could prevent the progression of PTC by querying CMap under the concept of reversing gene expression signatures.

## 2. Results

**Figure 1:**
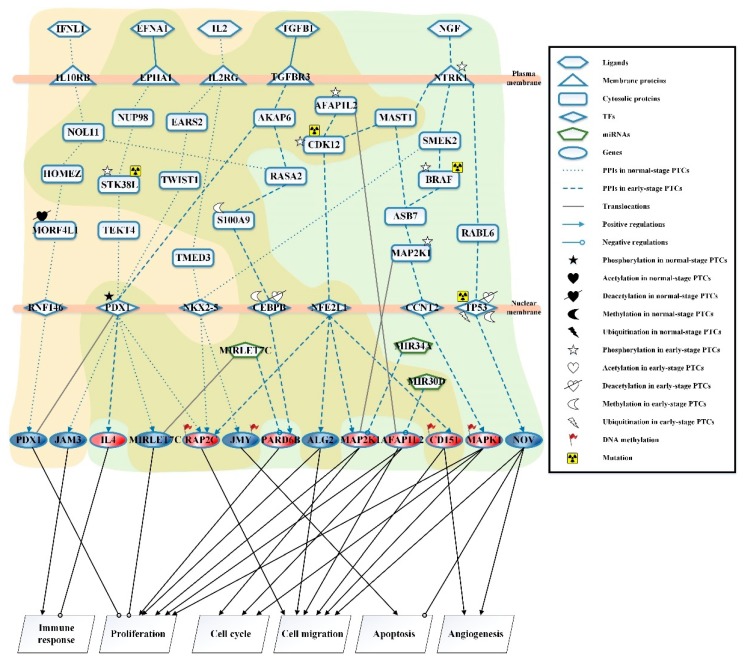
The core signaling pathways are obtained by projecting core GENs to KEGG pathways to investigate the carcinogenic progression mechanism from normal thyroid cells to early-stage papillary thyroid cancer cells. The blue dotted lines represent the signaling pathways in normal stage of thyroid cells; the blue dashed lines indicate the signaling pathways in early-stage of PTC; the blue solid lines denote the signal pathways in both stages; the gray solid lines represent translocation; the blue arrow head of lines represents upregulation; the blue circle head of lines represents downregulation; the black arrow head of solid lines represents activating cellular function; the black circle head of solid lines represents inhibiting cellular function; the red gene nodes indicate a higher gene expression in early-stage PTC cells compared with normal thyroid cells; the blue gene nodes indicate a lower gene expression in early-stage PTC cells compared with normal thyroid cells; the orange background covers the cellular molecules in normal stage of thyroid cells; the green background covers the cellular molecules in early-stage of thyroid cancer cells.

**Figure 2:**
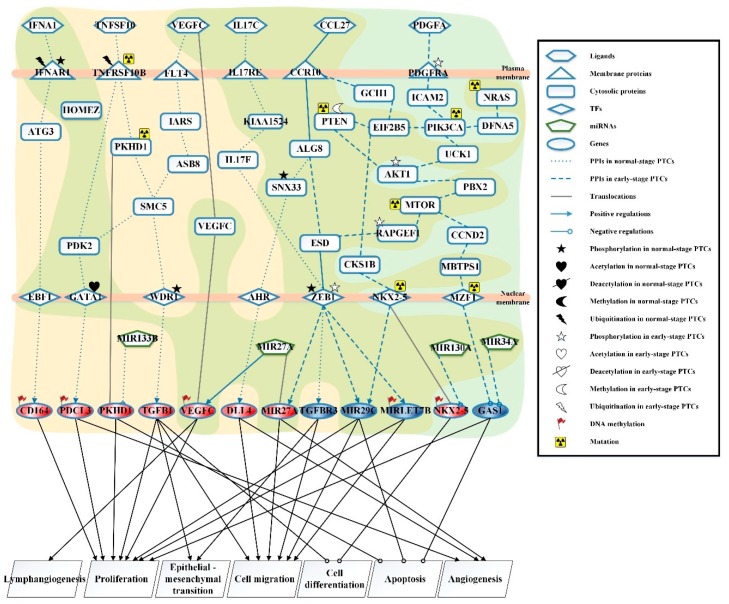
The core signaling pathways are obtained by projecting core GENs to KEGG pathways to investigate the carcinogenic progression mechanism from early-stage thyroid cancer cells to late-stage papillary thyroid cancer cells. The blue dotted lines represent the signaling pathways in early-stage of thyroid cells; the blue dashed lines indicate the signaling pathways in late-stage of PTC; the blue solid lines denote the signal pathways in both stage; the gray solid lines represent translocation; the blue arrow head of lines represents upregulation; the blue circle head of lines represents downregulation; the black arrow head of solid lines represents activating cellular function; the black circle head of solid lines represents inhibiting cellular function; the red gene nodes indicate a higher gene expression in late-stage PTC cells compared with early-stage cells; the blue gene nodes indicate a lower gene expression in late-stage PTC cells compared with early-stage PTC cells; the orange background covers the cellular molecules in early-stage of thyroid cancer cells; the green background covers the cellular molecules in late-stage of thyroid cancer cells.

The candidate GEN comprised candidate PPIN and candidate GEN. In this study, we utilized reverse engineering and system order detection methods on candidate GEN with corresponding normal, early, and late stage of PTC NGS data for obtaining real GENs shown in Figures S1–S3, respectively. The total number of nodes containing receptors, proteins, lncRNA, TFs and miRNA and edges of their interactions in candidate GEN and real GENs are shown in Table 1. It is noted that the number of receptors, proteins, lncRNA, TFs, and miRNA in candidate GENs decreases a lot compared to those in real GEN. This phenomenon demonstrated that the false-positives were eliminated by system identification and system order detection approaches. After applying the PNP method, we extracted core GENs from real GENs by selecting the top-ranked 2000 nodes with significant projection values that could reflect 85% of the real GENs in three stage of PTC, respectively. The higher the projection value is, the more contribution provided by the node in real GEN. Moreover, in order to be convenient for investigating the genetic and epigenetic molecular progression mechanism of PTC, we denoted core signaling pathway in respect of KEGG pathways. The differential core signaling pathways were distinguished and carcinogenic progression mechanisms were found for normal thyroid cells to early stage of PTC and early to late stage of PTC, shown in Figure 1 and Figure 2, respectively. Consequently, according to our analytic results, we identified two pools of essential biomarkers reflecting abnormal gene expression signatures for PTC progression. By querying CMap, we suggested six potential compounds with high negative connectivity scores for preventing progression from normal to early sate of PTC and early to late stage of PTC, respectively.

### 2.1. Investigating Carcinogenic Progression Mechanisms by Differential Core Signaling Pathways from Normal-Stage to Early-Stage Papillary Thyroid Cancer Cells

Based on the projection values of each element in core GENs, we selected and investigated the differential core signaling pathways from normal stage to early stage of PTC. In Figure 1, receptor IL10RB receives microenvironment factor IFNL1 (positive regulation signaling of immune response) to activate TF RNF146 through signaling transduction proteins NOL11, HOMEZ, and MORF4L1. The protein MORF4L1, which was affected by deacetylation, could promote the transmission of upstream signals to its transcription factor. TF RNF146 could positively regulate target gene *PDX1* to inhibit the proliferation of cancer cells. In the next two pathways of normal stage, the receptor EPHA1 could interact with ligand EFNA1 (cell–cell signaling) and receptor IL2RG could bind to ligand IL2 (T cell differentiation signaling) to trigger the TF PDX1 through the mediation of signaling transduction proteins NUP98, STK38L, and TEKT4, and signaling transduction proteins EARS2 and TWIST1, respectively. TF PDX1, which was affected by phosphorylation, could positively regulate *RAP2C*, *MIRLET7C*, and *JAM3*. Downstream target gene *RAP2C* could be affected by ligand EFNA1, and the lower gene expression of *RAP2C* could induce the inhibition of cell migration of thyroid cancer cells. The target genes *JAM3* and *MIRLET7C* could be regulated by the signal transduction pathways of ligand IL2 to cause the cellular immune response and inhibit the cell proliferation, respectively. STK38L was modified by phosphorylation and mutation to silence the signal transmission of EFNA1 that could cause truncation of this downstream signaling in early-stage of PTC. Besides, the microenvironment factor IL2 could trigger TF NKX2-5 through signaling transduction proteins TMED3 and TF NKX2-5 to positively regulate target genes *RAP2C* and *JMY*, which were modified by DNA methylation with a higher basal level in normal than early stage of PTC, and then the target gene *JMY* could promote tumor cells’ apoptosis.

For the core signaling pathways of early-stage thyroid cancer cells in Figure 1, the receptor TGFBR3 was activated by the transforming growth factor TGFB1 (cell development signaling) to modulate TF CEBPB to positively regulate gene *PARD6B*, which was also suppressed by *MIRLET7C*, to promote tumor cell proliferation through the mediation of signaling transduction proteins AKAP6, RASA2, and S100A9. Furthermore, S100A9 was affected by methylation and TF CEBPB was modified by deacetylation and methylation to silence this signaling pathway. Moreover, the signaling TGFB1 could also trigger TF PDX1 to positively regulate gene *IL4* to suppress the immune response through signaling transduction protein AKAP6. In the next pathways, the high affinity nerve growth factor receptor NTRK1, which was affected by phosphorylation, could trigger three TFs—NFE2L1, CCNT2, and TP53—by the modulation of three signaling pathways. NTRK1 could interact with ligand growth factor NGF (a signal of positive regulation of Ras protein signal transduction). In the first pathway, TF CCNT2 could positively regulate gene *MAPK1* through signaling transduction proteins SMEK2, BRAF, ASB7, and MAP2K1. Among these proteins, BRAF and MAP2K1 were affected by phosphorylation in early-stage thyroid cancer cells. Moreover, target gene *MAPK1* was modified by DNA methylation to promote PTC cell cycle, proliferation, and migration, and the mutation of protein BRAF was revealed in papillary thyroid cancer at the Cosmic database [25]. In the second pathway, the TF NFE2L1 could positively regulate *RAP2C*, *ALG2*, *MAP2K1*, and *CD151*, which was mediated by ligand NGF to activate receptor NTRK1 through signaling transduction proteins MAST1 and CDK12. Besides, the translocated target gene *AFAP1L2* was also suppressed by *MIR30D* to promote cell proliferation and migration; the target gene *RAP2C*, which was modified by DNA methylation with a higher basal level at equation (2) in Supplementary Materials 1.1, could induce cancer migration; the target gene *ALG2* could cause tumor cell proliferation and migration; the target gene *MAP2K1*, which was also suppressed by *MIR34A*, could lead to cancer cell proliferation and tumor cell cycle; the target gene *CD151* was affected by DNA methylation to promote thyroid cancer cell migration and angiogenesis. The signaling transduction proteins AFAP1L2 and CDK12 were modified by phosphorylation, and then phosphorylated AFAP1L2 could cause mutated CDK12 to conduct the signaling transduction in early-stage PTC. In the third pathway at the rightmost of Figure 1, TF TP53 was modified by deacetylation, ubiquitination, and methylation to inhibit signaling transduction through protein RABL6. TF TP53 was mutated to positively regulate target gene *NOV* to promote tumor cell migration, apoptosis suppression and angiogenesis.

By observing differential core signaling pathways in normal to early stage of PTC cells, cellular dysfunctions including immune response, proliferation, cell cycle, cell migration, apoptosis, and angiogenesis moved forward the progression of PTC. After querying CMap with the identified abnormal gene expression signature, we suggested six compounds—hydroxyachillin, sirolimus, meprylcaine, papaverine, boldine, and sulfamethoxypyridazine—to be our candidate drugs for avoiding progression from normal thyroid cells to early stage of PTC.

### 2.2. Investigating Carcinogenic Progression Mechanisms of Differential Core Signaling Pathways from Early-Stage to Late-Stage Papillary Thyroid Cancer Cells

The differential core signaling pathways from early-stage PTC to late-stage PTC are shown in Figure 2. For core signaling pathways in early-stage PTC, the interferon alpha/beta receptor IFNAR1, which was modified by phosphorylation and ubiquitination, could interact with ligand IFNA1 (cytokine-mediated signaling) to activate TF EBF1 through signaling transduction protein ATG3. TF EBF1 positively regulates the target gene *CD164* once modified by DNA methylation, which could promote proliferation of PTC cells. In the next pathway, the tumor necrosis factor receptor TNFRSF10B, which was affected by ubiquitination and mutation, receives tumor necrosis factor ligand TNFSF10 (a regulatory signal of extrinsic apoptotic signaling pathway) to trigger TF GATA1, which was affected by acetylation. TF GATA1 could positively regulate target gene *PDCL3*, which was modified by DNA methylation, to cause tumor cell proliferation and angiogenesis through signaling transduction protein PDK2. Moreover, signaling transduction protein PKHD1, which was mediated by receptor TNFRSF10B, could induce cancer cell proliferation and suppression of cancer cell apoptosis by the occurrence of genetic mutation and translocation. The target gene *PKHD1* was also suppressed by *MIR133B* in the cell nucleus. In the next core signaling pathway, the vascular endothelial growth factor receptor FLT4 could interact with ligand VEGFC (a positive regulation signaling of lymphangiogenesis) to trigger TF WDR1, which was affected by phosphorylation through signaling transduction proteins IARS, ASB8, and SMC5. TF WDR1 could positively regulate target gene *TGFB1* to promote cancer cell proliferation, epithelial–mesenchymal transition (EMT), and migration, and to suppress cancer cell differentiation. The signaling transduction protein VEGFC could be translocated to the nucleus in early-stage and late-stage PTC to promote lymphomagenesis and cancer cell proliferation. Besides, target gene *VEGFC* was inhibited by *MIR27A* and modified by DNA methylation in late-stage PTC. In the third core signaling pathway, the receptor IL17RE received ligand IL17C (an inflammatory response signaling) to trigger TF ZEB1, which was modified by phosphorylation by signaling transduction proteins KIAA1524 and IL17F. TF ZEB1 could positively regulate target gene *TGFBR3* to promote cancer cell EMT and migration. In the last core signaling pathway, ligand CCL27 (a signal of cell chemotaxis) could interact with receptor CCR10 to activate TF AHR through signaling transduction proteins ALG8 and SNX33, which were affected by phosphorylation. Moreover, TF AHR could positively regulate target gene *DLL4* to cause the migration and angiogenesis of thyroid cancer cells.

For core signaling pathways in late-stage PTC, the receptors CCR10 and PDGFRA could receive ligands CCL27 and PDGFA (a stimulation signal of inflammatory cytokines), respectively, to trigger TFs ZEB1, NKX2-5, and MZF1. Furthermore, PDGFRA was affected by phosphorylation that could activate TFs through three signaling pathways. In the first core signaling pathway, PDGFA/PDGFRA could trigger TF NKX2-5 through signaling transduction proteins ICAM2, PIK3CA, EIF2B5, and CKS1B. Furthermore, mutated NRAS could interact with DFNA5 to cause mutated PIK3CA become involved in this pathway. Then, mutated TF NKX2-5 could positively regulate target genes *MIR29C* and *MIRLET7B*. TF NKX2-5 could be translocated to the nucleus, which was negatively regulated by *MIR130A*. Therefore, the target gene *NKX2-5* could be modified by DNA methylation to suppress cancer cell differentiation. Moreover, the target gene *MIR29C* could promote cancer cell proliferation and migration, suppressing apoptosis; the target gene *MIRLET7B*, which was affected by DNA methylation, could cause the proliferation and migration of PTC. In the second core signaling pathway, PDGFA/PDGFRA could activate TF ZEB1, which was modified by phosphorylation, through signaling transduction proteins ICAM2, PIK3CA, UCK1, AKT1, PBX2, MTOR, RAPGEF1, and ESD. In this pathway, PTEN with deacetylation and mutation could promote the activation of mutated PIK3CA through EIF2B5 to interact with AKT1. Further, AKT1 and RAPGEF1 were affected by phosphorylation, and MTOR is mutated to regulate downstream proteins involved in oncogenesis and tumor invasion. In addition, CCL27/CCR10 could also trigger ZEB1 through signaling transduction proteins ALG8 and ESD. The activated TF ZEB1 could positively regulate target gene *MIR27A* to promote cancer cell migration and angiogenesis, and also positively regulate target gene *MIR29C* to promote cancer cell proliferation and migration and to suppress cancer cell apoptosis. The target gene *MIRLET7B* promotes tumor cell proliferation and migration after positive regulation by TF ZEB1. In the third core signaling pathway, PDGFA/PDGFRA could trigger the mutated TF MZF1 through signaling transduction proteins ICAM2, PIK3CA, UCK1, AKT1, PBX2, MTOR, CCND2, and MBTPS1. The mutated TF MZF1 could negatively regulate target gene *GAS1*, which was also suppressed by *MIR34A*, to cause the proliferation and suppress the apoptosis of thyroid cancer cells.

By observing differential core signaling pathways in the early to late stage of PTC cells, cellular dysfunctions, including lymphangiogenesis, proliferation, epithelial–mesenchymal transition, cell migration, cell differentiation, apoptosis, and angiogenesis, accelerated the progression of PTC. After querying CMap with an identified abnormal gene expression signature, we suggested six compounds—butyl hydroxybenzoate, morantel, fusidic acid, bephenium hydroxynaphthoate, sulconazole, and alclometasone—as candidates to prevent progression of PTC from early to late stage of PTC.

## 3. Discussion

Assisted with systems biology methods, differential carcinogenic progression mechanisms mediated by core signaling pathways of PTC cells are revealed. In normal stage to early-stage PTC cells, the core signaling pathways triggered by environmental factors influence target genes to bring about cellular dysfunctions, such as immune response, proliferation, cell cycle, cell migration, apoptosis, and angiogenesis, in the development of PTC. In early-stage to late-stage PTC cells, the core signaling pathways, as well as those induced by microenvironment changes, led to cellular dysfunctions such as lymphangiogenesis, proliferation, epithelial–mesenchymal transition, cell migration, cell differentiation, apoptosis, and angiogenesis, which play important roles in the progression of PTC. It is worth noting that essential biomarkers are found and two abnormal gene expression signatures are generated efficiently in systematic approaches. Ultimately, by querying CMap, we explored compounds with the potential be candidate drugs in future clinical trials.

### 3.1. The Carcinogenic Progression Mechanism from Normal Thyroid Cells to Early-Stage Papillary Thyroid Cancer Cells

In normal thyroid cells to early-stage PTC, as shown in Figure 1, microenvironment factor interferon lambda-1 IFNL1 (a positive regulation signaling of immune response) can inhibit cell proliferation and interact with receptor IL10RB through the crucial signaling pathway proteins NOL11, HOMEZ, and MORF4L1 to trigger RNF146. Besides, the ligand IFNL1 is mainly in epithelial tissues and has antitumor activity in the defense of the host. The signaling protein MORF4L1, involved in many diseases, has been reported to increase the homodimerization of MORF4L1, by the deacetylation of HDAC2-dependent MORF4L1 to promote complex formation to inhibit cell proliferation [26], and activate transcription factor RNF146 to positively regulate target gene *PDX1*, which is downregulated in the progression from normal cells to early-stage PTC cells. The overexpression of gene *PDX1* can suppress the proliferation of tumor cells and induce the apoptosis of cancer cells.

In the progression from normal to early stage PTC, the high concentration of ligand ephrin EFNA1 (cell–cell signaling) could reduce cell proliferation and cell migration speed. EFNA1 could also interact with ephrin receptor EPHA1, which is overexpressed in PTC and then associates with tumor growth, invasiveness, and metastasis, to trigger transcription factor PDX1, which is modified by phosphorylation to cause its activation [27]. The activated PDX1 by phosphorylation could upregulate target genes *MIRLET7C* and *RAP2C*. The gene *MIRLET7C* is a tumor suppressor gene, suppressing cancer cell proliferation in normal cells [28]. Our result shows that its expression declines over the course of carcinogenic progression. The overexpression of target gene *RAP2C* can promote cancer cell migration and invasiveness ability, but it has lower expression in normal thyroid cells compared with early-stage PTC cells. In addition, the mutation of the phosphorylation site of signaling transduction protein STK38L could ablate the kinase activity and block the activation, promoting cancer cell proliferation and destructing core pathway signaling transduction in the transforming early-stage thyroid cancer cells [29].

A classic Th1 cytokine IL2, expressed by activated T cells, could generate a new signal transduction pathway for gene transcription to enable cells to generate an effective antitumor immune response, interacting with cytokine receptor common subunit gamma IL2RG to activate transcription factors PDX1 and NKX2-5 by two different core pathways. In the first pathway, PDX1 could upregulate target gene *JAM3* to promote the cell immune response and inhibit tumor cell growth. Besides, the expression of *JAM3* is decreased in this progression and its defection may cause immunodeficiency in mice, which in turn reduces tumor immune surveillance [30]; in the second pathway, homeobox protein NKX2-5 upregulates target genes *PAP2C* and *JMY*, which can lead to cancer cell apoptosis. However, its expression is reduced in the carcinogenic progression of normal thyroid cells to early-stage PTC. Our result reveals that gene *JMY* has a higher basal level in normal thyroid cells due to DNA methylation level change.

The transforming growth factor TGFB1 (cell development signaling) is normally expressed and secreted by thyroid cells and shows a dual role in tumorigenesis, acting as a tumor suppressor in the early-stage of PTC [31]. The expression of TGFB1 is increased in tumor cells compared with normal thyroid tissue [32], and it could be accepted by transforming growth factor beta receptor TGFBR3, which is a tumor suppressor protein. When the signal is transmitted, the signaling transduction protein S100A9 is the target of the tumor-promoting signal, and it also activates downstream genes that promote tumor progression. However, S100A9 is modified by methylation so that its expression is silenced and upstream signals can be transmitted to the transcription factor CEBPB. TF CEBPB can upregulate target gene *PARD6B*, whose expression is increased to promote MAPK signaling pathway for tumor cell proliferation. In addition, transcriptional deactivation of CEBPB could induce the silence of the regulated gene *PARD6B* since it is affected by methylation and deacetylation [33]. Meanwhile, *MIRLET7C* could also inhibit the expression of *PARD6B* to cause inhibition of the proliferation of tumor cells. The ligand TGFB1 has an unfavorable effect on antitumor immunity, which significantly inhibits the immune surveillance toward host tumors [34] by prompting the downstream gene *IL4* expression to increase T cell polarization into Th2 cells. This results in the decline of Th1 cells and ultimately inactivates the tumor’s immune response.

NGF (a signal of positive regulation of Ras protein signal transduction) is a nerve growth factor that can bind to the tyrosine kinase receptor NTRK1 to induce phosphorylation and activate the following three core signal pathways, including the important mitogen-activated protein kinase MAPK signaling pathway.

In the first pathway, the signaling transduction protein BRAF, a carcinogenic factor, phosphorylates signaling transduction protein MAP2K1 when it is itself phosphorylated and mutated so that it remains active. Mutations in protein BRAF can affect the expression of noncellular components of tumor ECM (extracellular matrix) via MAPK signaling pathway to upregulate thyroid cancer microenvironmental regulator TSP1, thus changing the microenvironment in PTC [4]. The increased expression of *MAPK1*, which is upregulated by transcription factor CCNT2 and affected by DNA methylation, can lead to the growth and migration of tumor cells and initiate mitotic function to induce the cancer cell cycle. Especially, in a high iodine environment, *MAPK1* in PTC cells can also increase gene expression to speed cancer progress [35].

In the second pathway, the signaling transduction protein CDK12, which is activated by the signaling transduction proteins AFAPIL2 and MAST1, is a key regulator of transcription to maintain the genomic stability. When it phosphorylates to increase its own kinase activity to regulate transcription factors [36], its mutation activates TF NFE2L1, leading to the expression of downstream oncogenes. The multimodule structural adaptor protein AFAP1L2 can be involved in the regulation of signal transduction in various cells [37]. Besides, the activation of AFAP1L2 by phosphorylation can lead mutated CDK12 to promote tumorigenesis. When AFAP1L2 is translocated into the thyroid cell nucleus, it can promote cell cycle and induce cancer cells to inhibit apoptosis in early-stage PTC. *RAP2C* is overexpressed by TF NFE2L1 inducing cancer cell migration and invasiveness ability, it also has a higher basal level in early-stage PTC cells compared with normal thyroid cells, causing DNA methylation promoting gene activity and cell migration. The downregulation expression of tumor suppressor gene *ALG2* by TF NFE2L1 could inhibit cell proliferation and inactivate cell migration. The increased expression of *MAP2K1* is due to the positive regulation of NFE2L1 with phosphorylation, which could promote the progression of the tumor cell cycle and increase the growth and proliferation of thyroid cancer cells. In addition, *MIR34A*, which is a crucial regulator of tumor suppressor and is upregulated during early-stage PTC [38], may inhibit *MAP2K1*. However, the expression level of *MAP2K1* is still greater than *MIR34A*, and we deduce this might enhance cancer progression. *CD151* is involved in cancer progression and neovascularization, and its overexpression is upregulated by NFE2L1 and influenced by DNA methylation to cause a high migration ability of cancer cells in early-stage PTC. This is the key factor to tumor cell invasion, as long as *CD151* reduces the association with integrin that could reduce the invasion and metastasis of thyroid cancer cells [39].

In the third pathway, through the signaling transduction of ligand NGF signals, TF TP53, which could mediate cellular processes of cell apoptosis and migration, could inhibit the self-transcriptional activity by ubiquitination, deacetylation, and methylation, allowing the loss of original tumor suppressor function to increase tumor progression [40,41]. Moreover, the TP53 mutation can lead to impaired protein function inducing the genome instability that could positively regulate target gene *NOV*. *NOV* belongs to the CCN family and is downregulated in PTC cells. There is a literature supporting the hypothesis that the downregulation of *NOV*, which is a top androgen-repressed gene, can mediate the induction of androgens [42]. Therefore, we infer that the rise of androgens may enhance estrogen induction via synergy. Estrogen is a potent growth factor for benign and malignant thyroid cells, and it could stimulate angiogenesis and cell migration in cancer cells. Besides, the nuclear transcription factor ERβ of estrogen loses its expression resulting in the inhibition of apoptosis signaling causing the inhibition of apoptosis in cancer cells [3].

### 3.2. The Carcinogenic Progression Mechanism from Early-Stage to Late-Stage Papillary Thyroid Cancer Cells

According to the core signaling pathways of early-stage papillary thyroid cancer, shown in Figure 2, the ligand IFNA1 (a cytokine-mediated signaling) is a type I IFN pleiotropic cytokine, which can induce the activity of antitumor immune T cells and dendritic cells, and can directly inhibit the proliferation of normal tumor cells in vitro and vivo [43]. The receptor for IFNA1 is IFNAR1, which can induce phosphorylation through the P38 and PDK pathways and subsequent ligase-mediated ubiquitination to promote protein degradation [44] and trigger transcription factor EBF1 by signaling transduction protein ATG3. Activated EBF1 can upregulate target gene *CD164*, which is a cancer-promoting gene associated with tumorigenesis involved in the regulation of cell proliferation and adhesion. Besides, our results reveal that DNA methylation could cause *CD164* overexpression in the early stage of PTC, and it has been reported that *CD164* could promote the proliferation and metastasis of colon cancer cells in vivo and vitro through the CXCR4 pathway [45].

In the tumor necrosis factor TNF family, ligand member TNFSF10 (a regulation signal of extrinsic apoptotic signaling pathway) is mainly expressed in the cells of the immune system or oncogenic transformed cells, and it could induce apoptosis of thyroid cancer cells through interaction with the receptor TNFRSF10B [46]. However, it could produce resistance to the immune system to avoid cancer cells being destructed in the early-stage PTC. The receptor TNFRSF10B could cause degradation via ubiquitination [47] and trigger the transcription factor GATA1, and its mutation can enhance the suppression of apoptosis in thyroid cancer cells to promote the selective growth of cancer cells [48]. The acetylation of GATA1 could stimulate dependent transcriptional activation [49] to upregulate *PDCL3*, which mediates by DNA methylation simultaneously. The gene *PDCL3* is a chaperone molecule of VEGFR2, which can protect VEGFR2 from the ubiquitin-dependent degradation. The increased expression of gene *PDCL3* could significantly increase the ligand-stimulated phosphorylation of VEGFR2 and the downstream activation of key VEGFR2 [50]. In addition, the activated downstream of VEGFR2 can directly promote cancer cell proliferation and angiogenesis in vivo. PKHD1, which is mediated by receptor TNFRSF10B, mutates in PTC and induces expansion of the abnormality of the centrosome leading chromosome, which then leads to genome instability that could cause malignancy, promoting cancer cell proliferation and protecting cancer cells from apoptosis [51,52]. Furthermore, *MIR133B* was found to inhibit *PKHD1*, which suppresses the proliferation of thyroid cancer cells and promotes the death of cancer cells [53], but its expression is low in early-stage PTC.

In the next pathway, the effective lymphangiogenic factor VEGFC (a positive regulation signaling of lymphangiogenesis) could interact with receptor FLT4 and activate TF WDR1 via signaling transduction proteins IARS, ASB, and SMC5. *VEGFC*, which is promoted by *MIR27A*, could be translocated into the nucleus in the early-stage and late-stage of PTC. In addition, in the late-stage of PTC, *VEGFC* causes DNA methylation to increase and induces the proliferation of lymphatic endothelial cells and lymphoid hyperplasia in vivo [54]. Finally, it promotes lymph node metastasis to cause carcinogenic progression. TF WDR1 is a protein that could assist the actin-mediated disassembly of actin and it was identified as a key factor in the regulation of cell migration [55,56]. Besides, its phosphorylation could activate the self-expression to upregulate the expression level of *TGFB1*. *TGFB1* could act as a tumor promoter at the later stage of tumorigenesis, due to genetic and epigenetic changes. The cellular dysfunction of *TGFB1* may lead to tumor metastasis between the early and late stages of PTC [31]. Reports have pointed out that the persistent activation of TGFβ-mediated signaling pathways could promote EMT in mice [57] and increase thyroid tumor cell metastasis. Moreover, this signaling pathway could cause tumor cell metastasis that might induce the loss of differentiation of cancer cells. We speculate that this pathway could be one of the factors to convert a well-differentiated papillary thyroid cancer into a poorly differentiated thyroid cancer.

A cytokine IL17C (inflammatory response signaling) produced by lymphocyte subsets of Th17 cells is involved in the pathogenesis of chronic inflammatory responses and could induce the expression of vascular endothelial growth factor that could then induce TGFβ to cause tumor growth and metastasis [58]. In addition, the signaling is received by the receptor IL17RE and then is transduced to the transcription factor ZEB1 through signaling transduction proteins KIAA1524 and IL17F. TF ZEB1, which is an important regulator of EMT during embryonic development and cancer progression [59], is modified by phosphorylation to increase its transcriptional activity to downregulate the expression of *TGFBR3* and integrate cytokines and growth factors in the local environment of cancer cells [60]. *TGFBR3* is a tumor suppressor gene, which was found to decrease its expression to promote cancer cell migration and invasion [61,62]. Furthermore, compared with well-differentiated tumors, poorly differentiated tumors have a low expression of *TGFBR3*, suggesting that PTC may progress to poorly differentiated thyroid cancer in the late stage.

In the final core pathway of early-stage PTC, the chemokine ligand CCL27 (a signal of cell chemotaxis) is strongly upregulated by tumor necrosis factor (TNFα) and interleukin (IL1β), and is received by a specific receptor: CCR10. Furthermore, the signal transduction can increase the growth of tumor cells and invading tissues, thus evading the host immune response and spreading to lymph nodes [63]. When signaling to the novel protein SNX33, which is involved in endocytic transport, it is modified by phosphorylation to activate transcription factor AHR. Besides, AHR could regulate cellular functions of cell cycle, apoptosis, remodeling of extracellular matrix, and angiogenesis [64]. The activated TF AHR can upregulate the expression of target gene *DLL4* and activate the Notch signaling pathway, which can lead to tube formation in cancer cells, increase blood vessel branching, reduce pericyte recruitment, and reduce fibronectin expression [65]. Moreover, gene *DLL4* is closely related to lymph node metastasis and was found to be an independent predictor of lymph node metastasis of PTC. Additionally, *DLL4* could promote the differentiation of Th17 cells [66] and activate Th17 cellular functions to produce IL17, which can stimulate the mediation of core pathway by IL17C.

Focusing on the late-stage PTC core pathways identified in Figure 2, platelet-derived growth factor PDGFA and platelet-derived growth factor receptor PDGFRA are overexpressed in thyroid cancer. The expression of PDGFA is affected by the stimulation of inflammatory cytokines [67]. In addition, the autocrine activation of PDGFA is considered to play a key role in carcinogenesis. On the other hand, PDGFRA can cause cytoskeletal rearrangement, increasing migration potential, the formation of invasive pseudopodia, and EMT-mediated lymphatic metastasis in thyroid cancer cells [68]. Besides, PDGFRA ligand binding induces its dimerization, causing phosphorylation of specific tyrosine residues and subsequent recruitment of various signal transduction molecules, which could lead to the activation of the PI3K signaling pathway [69,70]. In our result, ligand PDGFA is received by receptor PDGFRA to mediate three core pathways and activate downstream cascade signaling proteins to three transcription factors ZEB1, NKX2-5, and MZF1, respectively.

In the first pathway in late-stage PTC, the receptor PDGFRA, which is modified by phosphorylation, interacts with PDGFA to mediate homeobox protein NKX2-5 through cascade signaling proteins ICAM2, PIK3CA, EIF2B5, and CKS1B. In this pathway, the mutation of NRAS [71] causes the mutation and amplification of PIK3CA [72], which can control cell proliferation, survival, and movement and many other cellular processes [73]. Furthermore, mutated PIK3CA could regulate TF NKX2-5 and induce the mutation of NKX2-5 via EIF2B5 and CKS1B, and then the mutated NKX2-5 is translocated into the nucleus and is regulated by *MIR130A*. The target gene *NKX2-5* is up-regulated in the nucleus due to DNA methylation, and its overexpression promotes the proliferation of cancer cells to induce the inhibition of cancer cell differentiation during thyroid carcinogenesis. By the way, the overexpression of *NKX2-5* could increase the production of hydrogen peroxide and promote the carcinogenic progression of thyroid cancer [74,75]. *MIR130A*, which could inhibit the gene *NKX2-5*, could act as a miRNA of tumor-inhibiting to inhibit cancer cell proliferation, migration and dedifferentiation. However, it is significantly downregulated in PTC [76].

In the second pathway of late-stage PTC, the receptor PDGFRA receives ligand PDGFA to mediate TF ZEB1 through signaling protein cascades ICAM2, PIK3CA, UCK1, AKT1, PBX2, MTOR, RAPGEF1, and ESD. In this pathway, mutated PIK3CA is promoted by NARS mutation and is also affected by mutated PTEN. Besides, the tumor suppressor protein PTEN is a dual protein and lipid phosphatase, its methylation and mutation can impair protein function, decrease expression, and promote PIK3CA signaling transduction [73,77]. Then, when the signal is transmitted to the protein AKT1, which is a key signaling intermediate for growth and survival, it is overactivated and overexpressed by phosphorylation [78] and the loss of PTEN activity [79], resulting in the activation of the downstream protein PBX2. Next, activated PBX2 signals to the mutated protein MTOR. The mammalian target of rapamycin MTOR is an important mediator of signal transduction to regulate cell growth, apoptosis and metabolism [80]. In addition, MTOR is overactivated in tumors to induce mutations and then mediate the phosphorylation of protein RAPGEF1. Phosphorylated RAPGEF1, which is involved in a variety of signaling mediators for cell adhesion, proliferation, and actin reorganization [81], could activate its expression and mediate signaling transduction protein ESD. ESD is mediated not only by PDGFA/PDGFRA pathway but also by CCL27/CCR10 pathway and it triggers TF ZEB1, which is modulated by phosphorylation. Phosphorylated ZEB1 could upregulate the expression of target gene *MIR27A*, which could enter the circulatory system through the cell matrix and blood vessel wall, causing migration, invasion, and angiogenesis in tissues. Moreover, *MIR27A* could also induce iNOS in tumor tissue, which may be one of factors causing the deterioration of cancer cells [82,83].

The transcription factors NKX2-5 and ZEB1, which are regulated by the first and second pathways of PDGFA/PDGFRA, respectively, positively regulate target genes *MIR29C* and *MIRLET7B* to cause their expression to decline. *MIR29C* as a tumor suppressor in PTC can downregulate oncogenes and upregulate tumor suppressors. In addition, its increasing expression can increase TP53 expression level to induce apoptosis and downregulate cyclin E expression to inhibit tumor growth [84]. The most important is that *MIR29C* can inhibit the AKT in the PI3K/AKT signaling transductance pathway to prevent the regulation of cellular functions by the cascade [9]. Target gene *MIRLET7B* is modified by DNA methylation to induce its expression inactivation that could upregulate the expression of cancer gene and subsequently cause transforming growth factor TGFβ/smad protein and Wnt/β-catenin signaling pathway to promote cancer cell proliferation and migration [85].

In the third pathway of late-stage PTC, ligand PDGFA interacts with its receptor PDGFRA to activate TF MZF1 through signaling transduction proteins ICAM2, PIK3CA, UCK1, AKT1, PBX2, MTOR, CCND2, and MBTPS1. In this pathway, mutated MTOR regulates cell proliferation and apoptosis, causing MZF1 transcription activation that can induce the downstream gene to have a similar cellular function. TF MZF1 is zinc finger protein family of transcription factors and its abnormal expression is an important factor that could interfere with hematopoietic cell proliferation and cell tumorigenesis [86]. Moreover, MZF1 is modified with mutation [87] to suppress the expression of gene *GAS1*. The silence of gene *GAS1* expression could activate RET to rise PI3K/AKT pathway activation to promote tumor cell proliferation and suppress apoptosis [88]. In addition, *MIR34A* can down-regulate *GAS1* expression level and its overexpression could promote cancer cell proliferation, stimulate colony formation and suppress cell apoptosis in late-stage papillary thyroid cancer.

### 3.3. The Overview of Genetic and Epigenetic Progression Mechanism From Normal Thyroid Cells to Late-Stage Papillary Thyroid Cancer Cells

**Figure 3:**
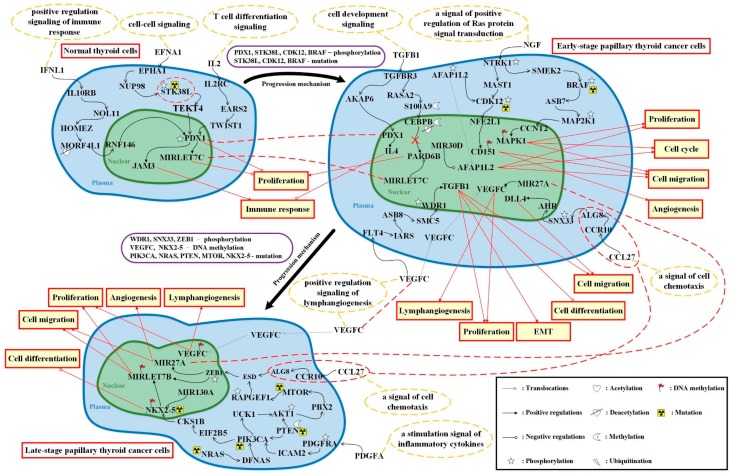
The overview of the proposed carcinogenic progression mechanism from normal to late-stage papillary thyroid cancer cells. This figure summarizes the genetic and epigenetic progression mechanisms of papillary thyroid cancer cells in Figure 1 and Figure 2. The upper horizontal part is the genetic and epigenetic progression mechanism from normal thyroid cells to early-stage papillary thyroid cancer cells; the top-right to bottom-left part denotes the genetic and epigenetic progression mechanisms from early-stage papillary thyroid cancer cells to late-stage papillary thyroid cancer cells; the red rectangle with yellow background represents cellular functions; the purple rounded rectangle indicates the biomarker to induce genetic and epigenetic progression mechanism of successive stages; the yellow dashed ellipse circles represent the microenvironment; the red dash lines denote biomarkers that appear in two consecutive stages of papillary thyroid cancer cells; the black arrow lines represent the protein–protein interaction or transcriptional regulation; the black circle head of lines indicates miRNA downregulation by miRNA; the red arrow lines represent the genes to induce cellular function; the red circle head of lines indicates the genes to repress cellular function.

The overall carcinogenic progression mechanisms are shown in Figure 3 by integrating the corresponding core signaling pathways in Figure 1 and Figure 2. The ligand signals are generated by the microenvironments to cause cellular dysfunctions that affect carcinogenic progression of PTC cells. It is noted that the genetic and epigenetic modifications of these core signaling pathways play key roles in causing cellular dysfunction.

In the normal thyroid cells shown in Figure 3, the ligand IFNL1 (positive regulation signaling of immune response) interacts with receptor IL10RB to upregulate target gene *PDX1*, which is overexpressed in the normal thyroid cells that can suppress tumor cell proliferation. Another ligand EFNA1 (cell–cell signaling) is received by receptor EPHA1 to trigger TF PDX1, which is modified by phosphorylation to cause activation [27], and to upregulate target gene *MIRLET7C*. Gene *MIRLET7C* is a tumor suppressor gene, suppressing cancer cell proliferation in normal cells [28]. The phosphorylation of protein STK38L could cause mutation to promote cancer cell proliferation and destruct the core signaling transduction pathway in early-stage thyroid cancer cells [29]. Next, ligand IL2 (a T cell differentiation signaling) binds to receptor IL2RC to trigger target gene *JAM3*, which could promote cell immune response and inhibit tumor cell growth.

In the early-stage PTC cells shown in Figure 3, ligand TGFB1 (a cell development signaling) binds to receptor TGFBR3 to mediate two following signaling pathways. It shows a dual role in tumorigenesis and acts as a tumor suppressor in the early-stage of PTC [31]. Due to the transcriptional activation and decreased expression of TF PDX1 in this stage, gene *IL4* expression is increased to suppress cellular immune responses. In addition, TF CEBPB is modified by methylation and deacetylation to cause silencing of downstream gene *PARD6B* [33]. Further, *MIRLET7C* could also inhibit the expression of *PARD6B*. Next, the ligand NGF (a signal of positive regulation of Ras protein signal transduction) interacts with NTRK1 to induce the MAPK signaling pathway causing the overexpression of target genes *CD151* and *MAPK1* by DNA methylation. The signaling protein BRAF is a carcinogenic factor, which is phosphorylated and mutated to cause expression to maintain its activity to activate target gene *MAPK1* to induce cancer cells’ proliferation, migration and cell cycle. The phosphorylation of protein CDK12 could cause mutation to dysregulate its transcriptional regulation. Besides, it could promote target gene *CD151* to induce tumor cell migration and angiogenesis [39].

In the following, we are going to discuss the carcinogenic progression from early-stage to late-stage PTC cells. In early-stage PTC cells, the receptor CCR10 is a selective chemical attractant to receive ligand CCL27 (a signal of cell chemotaxis), activating target gene *DLL4*, which could cause Notch signaling pathway and lymph node metastasis [65,66]. Ligand VEGFC (a positive regulation signaling of lymph angiogenesis) could interact with receptor FLT4 to upregulate target gene *TGFB1*, which is a tumor promoter in this carcinogenic progression to induce cancer cell proliferation, migration and EMT. Other than these, the most important is that it can suppress cell differentiation to let well differentiating PTC convert to poorly differentiating thyroid cancer cells [31,57]. Furthermore, VEGFC can be translocated to cell nuclear causing cancer cell proliferation and lymphangiogenesis through the promotion by *MIR27A* [54]. By the way, target gene *VEGFC* is modified by DNA methylation to cause overexpression and the overexpression of miRNA *MIR27A* could induce tumor cell migration and angiogenesis in late-stage PTC cells [82,83].

In late-stage PTC cells shown in Figure 3, the ligand PDGFA (a stimulation signal of inflammatory cytokines) is received by receptor PDGFRA to trigger PI3K signaling pathway [69,70]. Protein NRAS is modified by mutation to promote signaling protein PIK3CA mutation and amplification [71,72]. In addition, PTEN is a tumor suppressor and is modified by methylation and mutation to induce the reduction of its expression, thus boosting the activity of the downstream PIK3CA and AKT1 [73,77,79]. TF NKX2-5 is also affected by mutation to upregulate target gene *MIRLET7B*, which is modified by DNA methylation with the help of phosphorylated TF ZEB1, simultaneously. Besides, NKX2-5 translocates to nuclear and is modified by DNA methylation that could cause poor differentiation of thyroid cancer cells [74,75]. Further, *MIR130A* could inhibit *NKX2-5* to reduce cancer cell proliferation, migration and dedifferentiation, but it is significantly downregulated in thyroid cancer cells [76]. Finally, we find out some multiple drugs for the therapeutic treatment of carcinogenic progression of normal thyroid cells to late-stage PTC cells. More detailed analyses will be given in next section.

### 3.4. Exploring Multiple-Molecule Drugs for Papillary Thyroid Cancer by Querying the Connectivity Map

The systems biology approaches we proposed in this study make us identify essential biomarkers with specific gene expression signature efficiently from normal to early stage and early to late stage of PTC, respectively. Moreover, the biomarkers we found in two gene expression signatures not only have higher projection values but also express abnormally compared with normal thyroid cancer cells and early stage thyroid cancer cells. The goal of finding potential multiple-molecule drug is to explore compounds which could alter the identified gene expression signature. Notably, it is improper gene expression signature to move forward the progression from normal to early stage and early to late stage of thyroid cancer. Here, we applied CMap to analyze the aberrant gene expression signature. A high positive connectivity score indicates that the corresponding perturbagen induced the expression of the query signature. In other words, a high negative connectivity score indicates that the corresponding perturbagen reversed the expression of the query signature [89]. Therefore, to prevent the deterioration of PTC, we would choose compounds holding high negative connecting score to be our recommended candidate drugs.

In core signaling pathways of carcinogenic progression from normal thyroid cells to early-stage thyroid cancer cells shown in Figure 1, identified gene expression signature containing three abnormal downregulated genes—PDX1, JAM3, and NOV—and six abnormal upregulated genes—IL4, RAP2C, PARD6B, MAP2K1, CD151, and MAPK1. The chosen gene expression signature leads to dysfunction including immune response, proliferation, cell cycle, cell migration, apoptosis, and angiogenesis, which are affected by microenvironment changes and epigenetic modifications. After conducting CMap analysis, there are six compounds—hydroxyachillin, sirolimus, meprylcaine, papaverine, boldine, and sulfamethoxypyridazine—with high negative connectivity score having the ability to reverse the expression of our query signature. These suggested candidate compounds have the potential to prevent normal thyroid cells from transferring to early stage thyroid cancer cells. In the meantime, it is worthwhile noting that sirolimus in the combination with cyclophosphamide, ClinicalTroals.gov identifier NCT03099356, has been in the phase 2 clinical trial based on definitions developed by the U.S. Food and Drug Administration (FDA) for the treatment of metastatic differentiated thyroid cancer.

In core signaling pathways of carcinogenic progression from early to late stage of thyroid cancer cells shown in Figure 2, identified gene expression signature consisting of two abnormal downregulated genes—GAS1 and TGFBR3—and five abnormal upregulated genes—VEGFC, CD164, PDCL3, TGFB1, and NKX2-5. The chosen gene expression signature contributes to dysfunction, including lymphangiogenesis, proliferation, epithelial mesenchymal transition, cell migration, cell differentiation, apoptosis, and angiogenesis, which are triggered by microenvironment and modified by epigenetic modifications on the corresponding core signaling pathways. After conducting CMap analysis, there are six compounds—butyl hydroxybenzoate, morantel, fusidic acid, bephenium hydroxynaphthoate, sulconazole, and alclometasone—with high negative connectivity score having the ability to reverse the expression of our query signature. Suggested candidate compounds hold high probability to avoid switching from early stage thyroid cancer cells to late stage thyroid cancer.

### 3.5. The Limitations of Systems Biology Approches to Infer the Core Signaling Pathwayas of PTC

Along with the rapidly decreasing costs of molecular measurements, human beings enable not only profiling of disease sample molecular features at different levels (transcriptome, proteome and metabolome) but also measuring cellular signatures of individual drug in some clinically relevant models. Among molecular features, gene expression is the most widely used feature and has been extensively explored for drug target identification. In this study, in order to investigate the complex genetic and epigenetic progression molecular mechanisms of PTE, we leveraged genome-wide NGS data to construct GWGENs by the least square estimation method. Afterwards, we applied Akaike’s information criterion (AIC), a system order detection scheme, to do model selection. Even though AIC could help us to prune the false-positives of regulations and interactions in the GWGENs and conquer the overfitting problem, it is noted that the estimated model of GWGENs is a near-optimum solution, but not a unique solution. Moreover, the basal level parameters in systematic models of PPI, GRN, miRNA, and lncRNA, shown in Supplementary Materials 1.1, denote some unknown interactions which have not been considered in interaction equations such as epigenetic modifications and mutation. By observing a basal level change, which was higher than a threshold, we inferred the corresponding elements that were affected by epigenetic modifications or mutation, accompanied with a literature survey to validate our findings. Consequently, although the selected biomarkers have not been in the process of clinical trial, systems biology approach-based analyses in this study might provide new perspectives of understanding the progression molecular mechanisms of PTC in the system level and give an alternative way to speed up systems drug discovery for new therapeutics of PTC.

## 4. Materials and Methods

### 4.1. Overview of Constructing Candidate GEN and Core GENs in Normal Thyroid Cells and PTC Cells

**Figure 4:**
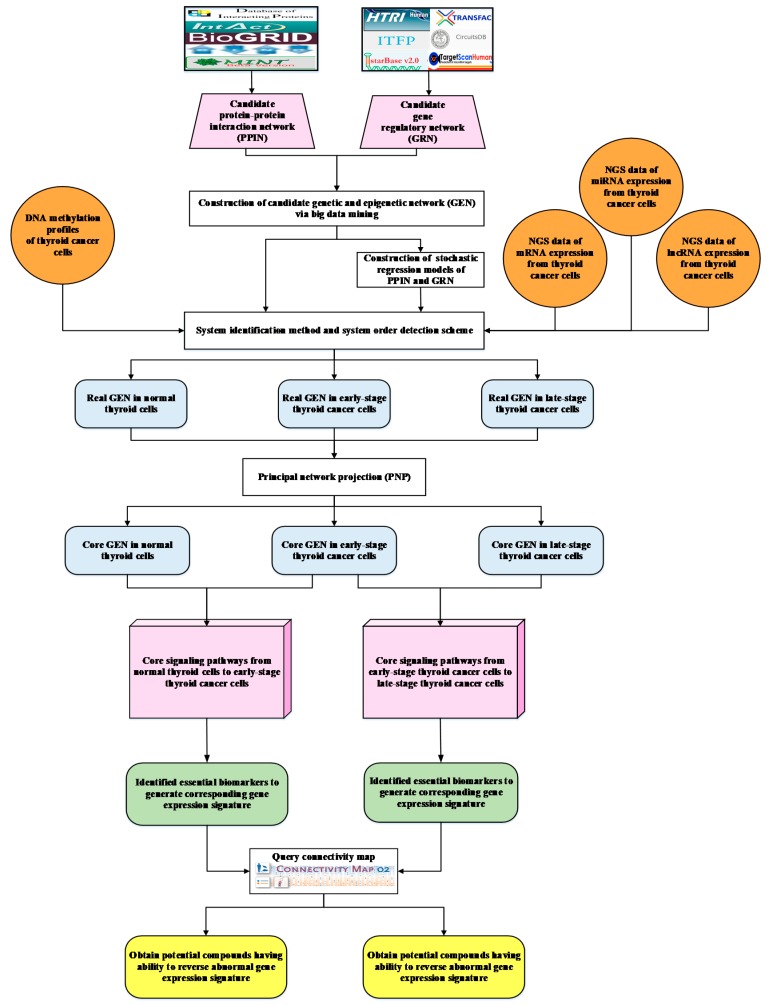
Flowchart of using systems biology methods to construct candidate genetic and epigenetic network (GEN), real GENs, core GENs, and core signaling pathways of carcinogenic progression mechanism in each stage of papillary thyroid cancer (PTC) for exploring drug combinations by querying CMap based on identified abnormal gene expression signatures. The pink trapezoid blocks represent the candidate protein–protein interaction network (PPIN), which was mined by databases DIP, IntAct, BioGRID, BIND, and MINT, and candidate gene, miRNA, and lncRNA regulatory networks (GRN), which were mined by databases HTRIdb, ITFP, StarBase2.0, TRANSFAC, CircuitDB, and TargetScanHuman; The orange round blocks denote NGS data of DNA methylation profile, mRNA, miRNA, and lncRNA. The white rectangular blocks indicate the methods applied to construct real GENs and extract core GENs. The blue rounded rectangular blocks are the real GENs and core GENs in normal thyroid cells, early-stage PTC cells, and late-stage PTC cells. The pink cuboid blocks represent core signaling pathways of two consecutive stages from normal thyroid cells to early-stage PTC cells and from early-stage to late-stage PTC cells. The green rounded rectangular blocks denote the identified gene expression signatures consisting of essential biomarkers lead to the progression of PTC. The following white rectangular block represents the querying CMap approach. The yellow rounded rectangular blocks show potential compounds after querying CMap based on our identified gene signatures.

To investigate the carcinogenic progression mechanisms of PTC, a flowchart is given in Figure 4. First of all, we conducted database mining to integrate regulation and interaction databases including DIP, IntAct, BioGRID, BIND, MINT, HTRIdb, ITFP, Transfac, Circuits DB2, and TargetScan for building candidate GEN represented by Boolean matrix (e.g., 0 or 1 if interaction is existent or nonexistent between two nodes). It is noted that normal thyroid cells, early and late papillary thyroid cancer cells share the same candidate GEN. The detailed procedure about system modeling for protein interaction, gene regulatory, miRNA regulatory, and lncRNA regulatory model could be found in Supplementary Materials 1.1. Secondly, after doing system modeling via genome-wide NGS data, we evaluated system model parameters by system identification. Due to various experimental conditions might lead to error within data coming from different database, we used system order detection scheme (Supplementary Materials 1.2) to prune false positives for obtaining real GENs of normal thyroid cells, early and late thyroid cancer cells which shown in Figures S1–S3. Thirdly, since real GENs are still too complicated to analyze, we extracted three core GENs from real GENs by PNP methods (Supplementary Materials 1.3) and denoted core signaling pathways in respect of KEGG pathways. Moreover, the core GENs are shown in Figures S4–S6. By doing so, based on the projection values, it would be easier to analyze and compare core signaling pathways within two consecutive stages (i.e., from normal thyroid cells to early-stage PTC cells and early-stage PTC cells to late-stage PTC cells). The identified essential biomarkers belonging to normal thyroid cells to early-stage PTC and early-stage to late-stage PTC are composed of two gene expression signatures, respectively. Eventually, we suggested potential compounds that could reverse the identified abnormal gene expression signatures to avoid PTC progression by querying CMap.

### 4.2. Genome-Wide NGS Data Preprocessing for Constructing GENs

We downloaded genome-wide mRNA (TCGA_THCA_exp_HiSeqV2_exon), miRNA (TCGA_THCA_miRNA_HiSeq), and DNA methylation profiles (TCGA_THCA_hMethyl450) NGS data from the Cancer Brower website (https://genome-cancer.ucsc.edu/). In this study, the clinical data of thyroid cancer include 65 samples of normal thyroid cells (Solid Tissue Normal), 213 samples of stage I PTC cells (Primary Tumor), 29 samples of stage II PTC cells (Primary Tumor), 72 samples of stage III PTC cells (Primary Tumor), and 45 samples of stage IV PTC cells (Primary Tumor), which are sorted out by The Cancer Genome Atlas (TCGA). In this dataset, due to fewer samples in stage II and stage IV, we grouped stage I and stage II as early-stage and stage III and stage IV as late-stage. Then, we constructed the candidate GEN by big database mining: candidate protein–protein interactions (PPIs) were mined from DIP, IntAct, BioGRID, BIND, and MINT; transcriptional factors (TF) and their target genes were extracted from ITFP, HTRIdb, Circuits DB2; and miRNAs and their target genes were extracted from TargetScan. According to our analyses, the GEN contains 4,285,416 candidate PPIs, 227 candidate regulations between TFs and lncRNAs, 2488 candidate regulations between TFs and miRNAs, 87,257 candidate regulations between TFs and genes, 819 candidate regulations between miRNAs and lncRNAs, 172 candidate regulations between miRNAs and miRNAs, and 220,494 post-transcriptional regulations between miRNAs and genes as shown in Table 1.

## 5. Conclusions

The proposed systems biology approaches, including system modeling, system identification, system model selection, and principal network projection methods, have been used to analyze and interpret heterogeneity data. In these ways, the core genome-wide genetic and epigenetic progression networks for each stage of PTC were identified from complex models of interaction and regulatory networks. Considered microenvironmental contributions, the identified core signaling networks demonstrate how they receive, transmit and integrate signaling resulting in various molecular mechanisms which provide us an opportunity to understand the progression of PTC in panoramic view. Moreover, the pathophysiological investigation of the PTC through the identified core networks could be applied in the clinic to improve the diagnosis and the administration of mechanism-driven interventions. Meanwhile, based on the ranking from principal network projection method, characterized essential components could be selected as candidate drug targets for multiple molecular drug design by CMap to prevent the progression of PTC. By transforming biology knowledge into systematic interpretation, systems biology approaches applied to medical practice is the future of systems medicine.

## Figures and Tables

**Table 1:** The total number of nodes and edges in candidate GENs and identified GENs at each stage of PTC.

Nodes/Edges	Candidate GEN	Normal GEN	Early GEN	Late GEN
**TF-lncRNA**	227	114	110	113
**TF-miRNA**	2488	1626	1945	1839
**TF-gene**	87,257	46,800	46,618	46,317
**TF-TF**	30,970	17,149	15,263	14,632
**TFs**	2122	1590	1646	1611
**lncRNA-lncRNA**	4	0	0	0
**lncRNA-miRNA**	0	0	0	0
**lncRNA-gene**	832	108	111	87
**lncRNA-TF**	322	67	75	64
**lncRNAs**	197	103	114	115
**miRNA-lncRNA**	819	95	158	134
**miRNA-miRNA**	172	22	15	15
**miRNA-gene**	220,494	34,858	33,447	34,389
**miRNA-TF**	45,908	7219	3833	3688
**miRNAs**	999	829	805	833
**Receptors**	2583	2376	2433	2448
**PPIs**	4,285,416	525,333	856,115	950,194
**Proteins**	14,583	13,602	13,789	13,806
**Total Nodes**	20,484	18,500	18,787	18,813
**Total edges**	4,674,909	633,391	957,690	1,051,472

Candidate GEN represents candidate genetic and epigenetic network; Normal GEN denotes identified GEN of normal thyroid cells; Early GEN indicates identified GEN of early-stage papillary thyroid cancer cells; Late GEN represents identified GEN of late-stage papillary thyroid cancer cells; TF-lncRNA, TF-miRNA, TF-gene, and TF-TF represent transcriptional regulation of lncRNA, miRNA, genes, and TF by TFs, respectively; lncRNA-lncRNA, lncRNA-miRNA, lncRNA-gene, and lncRNA-TF indicate transcriptional regulation of lncRNA, miRNA, gene, and TF by lncRNAs, respectively; miRNA-lncRNA, miRNA-miRNA, miRNA-gene, and miRNA-TF denote post-transcriptional regulation of lncRNA, miRNA, gene, and TF by miRNAs, respectively; TFs, lncRNAs, miRNAs, Receptors, and Proteins represent total number of TF, lncRNA, membrane protein, and protein.

## Supplementary Materials

Supplementary materials can be found at https://www.mdpi.com/1422-0067/20/10/2536/s1.
