# Supplementary material for: Systems Biology Approaches to Investigate Genetic and Epigenetic Molecular Progression Mechanisms for Identifying Gene Expression Signatures in Papillary Thyroid Cancer
Source: Int J Mol Sci. 2019 May 23;20(10):2536. doi: 10.3390/ijms20102536 (PMC6566633; doi:10.3390/ijms20102536)
Supplement: Supplementary file 1 [file ijms-20-02536-s001.pdf]

Supplementary Materials

1.1 Constructing real PPINs and real GRNs in real GENs at different stages of PTC cells

In order to obtain the real GENs in normal thyroid cells, early-stage, and late-stage PTC shown in Figures S1-S3, we used system identification and system order detection method prune false-positives of candidate GEN by their corresponding NGS data.

For the protein interactive systematic model of candidate PPIN in candidate GEN, the protein interactions of the *s*th protein in thyroid cells for sample *n* are described by the following protein interactive equations:

$$y_s[n] = \sum_{\substack{g=1 \\ g \neq s}}^{S_s} \alpha_{sg} y_s[n] y_g[n] + b_s + v_s[n], \text{ for } s=1, \ldots, S \text{ and } n=1, \ldots, N \quad (1)$$

where $y_s[n]$ denotes the expression level of the *s*th protein and $y_g[n]$ denotes the expression level of the *g*th protein for the *n*th sample; α_{sg} is the interaction ability between the *s*th protein and the *g*th protein; b_s represents the basal level of protein *s*, which is used to model the unknown interactions like acetylation, ubiquitination etc.; $v_s[n]$ is the stochastic noise of the *s*th protein for the sample *n*, which is due to data noise and model uncertainty; S_s represents the number of proteins interacting with the *s*th protein; and *S* represents number of proteins in candidate PPIN; *N* represents the number of data samples (patients).

For the gene regulatory systematic model of candidate GRN in candidate GENs, the transcriptional regulation of the *i*th gene of thyroid cells in sample *n* is given in the following equation:

$$x_i[n] = \sum_{s=1}^{S_i} \beta_{is} y_s[n] M_i[n] + \sum_{t=1}^{T_i} \tau_{it} l_t[n] M_i[n] - \sum_{w=1}^{W_i} \delta_{iw} x_i[n] r_w[n] M_i[n] + k_i M_i[n] + \varepsilon_i[n] \ , \quad (2)$$

for i=1, …,I and n=1, …,N

where $x_i[n]$ denotes the expression level of the ith target gene, $y_s[n]$, l_t[n] and $r_w\,[n]$ are the expression levels of the sth TF, the tth lncRNA and the wth miRNA for the nth sample, respectively; β_{is} and τ_{it} are the transcription regulatory ability of the sth TF and the tth lncRNA on their ith target gene, respectively; δ_{iw} represents the post-transcriptional regulatory ability of the wth miRNA to inhibit the ith target gene ($-\delta_{ip} \leq 0$); k_i indicates the basal level of target gene i; $M_i\,[n]$ represents the effect of methylation regulation of the ith target gene on the binding affinities of TFs, lncRNAs and miRNAs [1-3]; $\varepsilon_i\,[n]$ is the stochastic noise of the ith target gene for the sample n, which is due to data noise and model uncertainty; S_i represents the total number of TFs on the ith target gene; T_i represents total number of lncRNA bindings to the ith target gene; W_i represents total number of miRNA bindings to the ith target gene; I indicates the number of genes; N indicates the number of data samples. The methylation regulation on the ith target gene $M_i\,[n]$ by DNA methylation profile $m_i[n]$ can be shown as follows [4]:

$$M_i[n] = \left(1 + \left(2m_i[n]\right)^2\right)^{-1} \tag{3}$$

where $m_i[n]$ denotes the DNA methylation profile of the ith gene for the sample n and we find this range of effect of DNA methylation profile is between 0 and 1; and the range of effect $M_i\,[n]$ of DNA methylation on target gene i is from 1 to 0.2. The meaning of equation (2) and (3) is that a lower DNA methylation expression level shows a stronger binding affinity between target genes and TFs, lncRNAs, miRNAs. On the other hand, a higher DNA methylation expression level shows a weaker binding affinity between target genes and TFs, lncRNAs, miRNAs. For example, in equation (3), the DNA methylation profile $m_i[n]$ value is close to 0, then the DNA methylation regulation level $M_i\,[n]$ is close to 1, i.e., the bindings of TFs and lncRNAs and the post-

transcriptional regulations of miRNAs are not influenced by DNA methylation in equation (2).

In candidate GRNs, the transcriptional expression level $r_p[n]$ of the pth miRNA regulatory model of thyroid cells for sample n is given in the following:

$$\begin{aligned} r_p[n] = &\sum_{s=1}^{S_p} \lambda_{ps} y_s[n] M_p[n] + \sum_{t=1}^{T_p} \mu_{pt} l_t[n] M_p[n] \\ &- \sum_{w=1}^{W_p} \psi_{pw} r_p[n] r_w[n] M_p[n] + e_p M_p[n] + \omega_p[n], \qquad (4) \\ &\text{for } p=1, \ldots, P \text{ and } n=1, \ldots, N \end{aligned}$$

where $r_w[n]$ represents the expression level of the wth miRNA for the nth sample; $y_s[n]$ represents the expression level of the sth TF for the nth sample; $l_t[n]$ represents the expression level of the tth lncRNA for the nth sample; e_p represents the basal level of pth target miRNA; $M_p [n]$ represents methylation regulation of the pth miRNA, which has effect on the binding affinities of miRNAs and TFs on the target miRNA; $\omega_p[n]$ denotes the stochastic noise of the pth miRNA for the sample n, which is due to data noise and model uncertainty; λ_{ps}, μ_{pt} and ψ_{pw} are the transcription regulatory ability of the sth TF on the pth miRNA, transcription regulatory ability of the tth lncRNA on the pth miRNA and the post-transcriptional regulatory ability of the wth miRNA to inhibit the pth miRNA, respectively; S_p indicates the total number of TF bindings to the pth miRNA; T_p indicates the total number of lncRNA bindings to the pth miRNA; W_p indicates the total number of miRNAs bindings to the pth miRNA; P and N represents the total number of miRNAs and data samples, respectively.

In addition, the transcriptional expression level $l_q[n]$ of the qth lncRNA regulatory model of thyroid cells for sample n in candidate GRNs, is given in the following:

$$l_q[n] = \sum_{s=1}^{S_q} \gamma_{qs} y_s[n] M_q[n] + \sum_{t=1}^{T_q} \rho_{qt} l_t[n] M_q[n]$$

$$-\sum_{w=1}^{W_q} \zeta_{qw} l_q[n] r_w[n] M_q[n] + f_q M_q[n] + \eta_q[n], \quad (5)$$

for q=1, ...,Q and n=1, ...,N

where $y_s[n]$, $l_t[n]$ and $r_w[n]$ represent the expression levels of the sth TF, the tth lncRNA and the wth miRNA for the nth sample, respectively; $M_q[n]$ denotes the methylation regulation of the qth lncRNA, which has the effect on the binding affinities of TFs and miRNAs on the target lncRNA; f_q denotes the basal level of the qth lncRNA; $\eta_g[n]$ represents the stochastic noise of the qth target lncRNA for the sample n due to data noise and model uncertainty; γ_{qs} is the transcription regulatory ability of the sth TF on binding the qth target lncRNA; ρ_{qt} is the transcription regulatory ability of the tth lncRNA on binding the qth target lncRNA; ζ_{qw} is the post-transcriptional regulatory ability of the wth miRNA to inhibit the qth target lncRNA; S_q denotes the total number of TFs; T_q denotes the total number of lncRNA bindings to the qth lncRNA; W_q denotes the total number of miRNAs bindings to the qth lncRNA; Q denotes the total number of lncRNAs; N denotes the total number of data samples.

1.2 Parameter estimation of the systematic models of candidate GENs via system identification method and system order detection

Before the obtainment of real GENs, we constructed the candidate protein-protein interaction network (PPIN) model (1) and gene, miRNA and lncRNA regulatory network models (2), (4), and (5) in candidate GRN, respectively; we use the system identification method and system order detection scheme to identify the protein interactive parameters α_{sg}, b_s in protein-protein interaction model and regulatory parameters β_{is}, τ_{it}, δ_{iw}, k_i, λ_{ps}, μ_{pt}, ψ_{pw}, e_p, γ_{qs}, ρ_{pt}, ζ_{qw}, f_q of gene, miRNA and lncRNA regulatory models by NGS data of each thyroid to prune the false positives of candidate

PPINs and GRNs to identify the real GENs of every stage of PTC. In order to identify these interactive and regulatory parameters, we can rewrite equations (1), (2), (4), (5) as the following linear regression forms:

$$y_s[n] = \begin{bmatrix} y_s[n]y_1[n] & \cdots & y_s[n]y_{S_s}[n] & 1 \end{bmatrix} \times \begin{bmatrix} \alpha_{s1} \\ \vdots \\ \alpha_{sS_s} \\ b_s \end{bmatrix} + v_s[n] \quad (6)$$

$$x_i[n] = \begin{bmatrix} y_1[n]M_i[n] & \cdots & y_{S_i}[n]M_i[n] & l_1[n]M_i[n] & \cdots & l_{T_i}[n]M_i[n] \\ x_i[n]r_1[n]M_i[n] & \cdots & x_i[n]r_{W_i}[n]M_i[n] & M_i[n] \end{bmatrix} \times \begin{bmatrix} \beta_{i1} \\ \vdots \\ \beta_{iS_i} \\ \tau_{i1} \\ \vdots \\ \tau_{iT_i} \\ -\delta_{i1} \\ \vdots \\ -\delta_{iW_i} \\ k_i \end{bmatrix} + \varepsilon_i[n] \quad (7)$$

$$r_p[n] = \begin{bmatrix} y_1[n]M_p[n] & \cdots & y_{S_p}[n]M_p[n] & l_1[n]M_p[n] & \cdots & l_{T_p}[n]M_p[n] \\ r_p[n]r_1[n]M_p[n] & \cdots & r_p[n]r_{W_p}[n]M_p[n] & M_p[n] \end{bmatrix} \times \begin{bmatrix} \lambda_{p1} \\ \vdots \\ \lambda_{pS_p} \\ \mu_{p1} \\ \vdots \\ \mu_{pT_p} \\ -\psi_{p1} \\ \vdots \\ -\psi_{pW_p} \\ e_p \end{bmatrix} + \omega_p[n] \quad (8)$$

$$l_q[n] = \Big[y_1[n]M_q[n] \ \cdots \ y_{S_q}[n]M_q[n] \ \ l_1[n]M_q[n] \ \cdots \ l_{T_q}[n]M_q[n] \quad l_q[n]r_1[n]M_q[n] \ \cdots \ l_q[n]r_{W_q}[n]M_q[n] \ \ M_q[n] \Big] \cdot \begin{bmatrix} \gamma_{q1} \\ \vdots \\ \gamma_{qS_q} \\ \rho_{q1} \\ \vdots \\ \rho_{qT_q} \\ -\zeta_{q1} \\ \vdots \\ -\zeta_{qW_q} \\ f_q \end{bmatrix} + \eta_q[n] \tag{9}$$

The above regression equations (6), (7), (8), (9) can be simply represented as the following regression forms, respectively:

$$y_s[n] = \phi_{s,P}[n] \cdot \theta_{s,P} + v_s[n], \text{ for } s=1, \ldots, S \text{ and } n=1, \ldots, N \tag{10}$$

$$x_i[n] = \phi_{i,G}[n] \cdot \theta_{i,G} + \varepsilon_i[n], \text{ for } i=1, \ldots, I \text{ and } n=1, \ldots, N \tag{11}$$

$$r_p[n] = \phi_{p,M}[n] \cdot \theta_{p,M} + \omega_p[n], \text{ for } p=1, \ldots, P \text{ and } n=1, \ldots, N \tag{12}$$

$$l_q[n] = \phi_{q,L}[n] \cdot \theta_{q,L} + \eta_q[n], \text{ for } q=1, \ldots, Q \text{ and } n=1, \ldots, N \tag{13}$$

where $\theta_{s,P}$ denotes the parameter vectors related to protein and protein interaction abilities; $\theta_{i,G}$, $\theta_{p,M}$, and $\theta_{q,L}$ denotes the parameter vectors related with gene, miRNA and lncRNA transcriptional regulations, which contain transcriptional regulatory abilities, post-transcriptional regulatory abilities and basal levels, respectively; $\phi_{s,P}[n]$, $\phi_{i,G}[n]$, $\phi_{p,M}[n]$, and $\phi_{q,L}[n]$ represent the regression vectors of the expression data of protein, gene, miRNA, lncRNA that were also regulated by DNA methylation profiles of the nth sample (patient), respectively.

Furthermore, if all N samples need to be employed for parameter estimation, then, these equations can be written in the followings:

$$\begin{bmatrix} y_s[1] \\ y_s[2] \\ \vdots \\ y_s[N] \end{bmatrix} = \begin{bmatrix} \phi_{s,P}[1] \\ \phi_{s,P}[2] \\ \vdots \\ \phi_{s,P}[N] \end{bmatrix} \theta_{s,P} + \begin{bmatrix} v_s[1] \\ v_s[2] \\ \vdots \\ v_s[N] \end{bmatrix} \tag{14}$$

$$\begin{bmatrix} x_i[1] \\ x_i[2] \\ \vdots \\ x_i[N] \end{bmatrix} = \begin{bmatrix} \phi_{i,G}[1] \\ \phi_{i,G}[2] \\ \vdots \\ \phi_{i,G}[N] \end{bmatrix} \theta_{i,G} + \begin{bmatrix} \varepsilon_i[1] \\ \varepsilon_i[2] \\ \vdots \\ \varepsilon_i[N] \end{bmatrix} \tag{15}$$

$$\begin{bmatrix} r_p[1] \\ r_p[2] \\ \vdots \\ r_p[N] \end{bmatrix} = \begin{bmatrix} \phi_{p,M}[1] \\ \phi_{p,M}[2] \\ \vdots \\ \phi_{p,M}[N] \end{bmatrix} \theta_{p,M} + \begin{bmatrix} \omega_p[1] \\ \omega_p[2] \\ \vdots \\ \omega_p[N] \end{bmatrix} \tag{16}$$

$$\begin{bmatrix} l_q[1] \\ l_q[2] \\ \vdots \\ l_q[N] \end{bmatrix} = \begin{bmatrix} \phi_{q,L}[1] \\ \phi_{q,L}[2] \\ \vdots \\ \phi_{q,L}[N] \end{bmatrix} \theta_{q,L} + \begin{bmatrix} \eta_q[1] \\ \eta_q[2] \\ \vdots \\ \eta_q[N] \end{bmatrix} \tag{17}$$

which could be simply represented as follows, respectively,

$$Y_s = \Phi_{s,P} \cdot \theta_{s,P} + W_{s,P} \tag{18}$$

$$X_i = \Phi_{i,G} \cdot \theta_{i,G} + W_{i,G} \tag{19}$$

$$R_p = \Phi_{p,M} \cdot \theta_{p,M} + W_{p,M} \tag{20}$$

$$L_q = \Phi_{q,L} \cdot \theta_{q,L} + W_{q,L} \tag{21}$$

We have to estimate the parameter vectors $\theta_{s,P}$, $\theta_{i,G}$, $\theta_{p,M}$ and $\theta_{q,L}$ by NGS data and DNA methylation profiles of each thyroid condition. By the least square estimation, we could estimate parameter vectors $\theta_{s,P}$, $\theta_{i,G}$, $\theta_{p,M}$, $\theta_{q,L}$ as follows:

$$\min_{\theta_{s,P}} \frac{1}{2} \left\| \Phi_{s,P} \cdot \theta_{s,P} - Y_s \right\|_2^2 \tag{22}$$

$$\min_{\theta_{i,G}} \frac{1}{2} \left\| \Phi_{i,G} \cdot \theta_{i,G} - X_i \right\|_2^2 \tag{23}$$

$$\text{subject to} \quad \begin{bmatrix} \overbrace{0 & \cdots & 0}^{S_i} & \overbrace{0 & \cdots & 0}^{T_i} & \overbrace{1 & \cdots & 0}^{W_i} & 0 \\ \vdots & \ddots & \vdots & \vdots & \ddots & \vdots & \vdots & \ddots & \vdots & \vdots \\ 0 & \cdots & 0 & 0 & \cdots & 0 & 0 & \cdots & 1 & 0 \end{bmatrix} \theta_{i,G} \le \begin{bmatrix} 0 \\ \vdots \\ 0 \end{bmatrix}$$

$$\min_{\theta_{p,M}} \frac{1}{2} \left\| \Phi_{p,M} \cdot \theta_{p,M} - R_p \right\|_2^2 \tag{24}$$

$$\text{subject to} \quad \begin{bmatrix} \overbrace{0 & \cdots & 0}^{S_p} & \overbrace{0 & \cdots & 0}^{T_p} & \overbrace{1 & \cdots & 0}^{W_p} & 0 \\ \vdots & \ddots & \vdots & \vdots & \ddots & \vdots & \vdots & \ddots & \vdots & \vdots \\ 0 & \cdots & 0 & 0 & \cdots & 0 & 0 & \cdots & 1 & 0 \end{bmatrix} \theta_{p,M} \le \begin{bmatrix} 0 \\ \vdots \\ 0 \end{bmatrix}$$

$$\min_{\theta_{q,L}} \frac{1}{2} \left\| \Phi_{q,L} \cdot \theta_{q,L} - L_q \right\|_2^2 \tag{25}$$

$$\text{subject to} \quad \begin{bmatrix} \overbrace{0 & \cdots & 0}^{S_q} & \overbrace{0 & \cdots & 0}^{T_q} & \overbrace{1 & \cdots & 0}^{W_q} & 0 \\ \vdots & \ddots & \vdots & \vdots & \ddots & \vdots & \vdots & \ddots & \vdots & \vdots \\ 0 & \cdots & 0 & 0 & \cdots & 0 & 0 & \cdots & 1 & 0 \end{bmatrix} \theta_{q,L} \le \begin{bmatrix} 0 \\ \vdots \\ 0 \end{bmatrix}$$

The constrained least square parameter estimation equations in (22), (23), (24), (25) could guarantee the estimated post-transcriptional regulatory abilities of miRNA on the regulatory genes, lncRNAs and miRNAs are always to be negative. Consequently, we could always use the constrained least square parameter estimation method to obtain protein interactive estimation parameters, $\hat{\theta}_{s,P}$ and gene, lncRNA and miRNA regulatory estimation parameters $\hat{\theta}_{i,G}$, $\hat{\theta}_{p,M}$ and $\hat{\theta}_{q,L}$ by MATLAB optimization toolbox.

In PTC, we could obtain the gene, lncRNA and miRNA expression level data and DNA methylation profiles at normal stage, early stage and late stage of PTC from some public information such as PTC cells database of TCGA and Cancer Brower website to obtain $\Phi_{s,P}$, $\Phi_{i,G}$, $\Phi_{p,M}$, $\Phi_{q,L}$, Y_s, X_i, R_p and L_q. Then by the constrained least square parameter estimation problems in (22), (23), (24), (25), we could identify the interactive abilities, regulatory abilities and basal levels in the corresponding estimated parameters

$\hat{\theta}_{s,P}$, $\hat{\theta}_{i,G}$, $\hat{\theta}_{p,M}$ and $\hat{\theta}_{q,L}$. Since there still exist a lot of false-positives from different databases or some experimental data, we apply system order detection method to prune false-positives protein interactive abilities, transcriptional regulatory abilities and post-transcriptional regulatory abilities from candidate GEN to obtain real GENs.

One of system order detection scheme, Akaike Information Criterion (AIC) could detect the system order (the number of regulations) of real GRN through the identification system scheme. The system order detection criteria AICs including the sth protein of protein interaction, the ith gene, the qth lncRNA and the pth miRNA are respectively shown as follows:

$$AIC(S_s) = \log(\hat{\sigma}^2_{s,P}) + \frac{2(\Delta_{s,P})}{N} \quad (26)$$

where $\hat{\sigma}^2_{s,P} = \left(Y_s - \left(\Phi_{s,P}\hat{\theta}_{s,P}\right)\right)^T \left(Y_s - \left(\Phi_{s,P}\hat{\theta}_{s,P}\right)\right) / N$, $\Delta_{s,P} = S_s+1$

$$AIC(S_i, T_i, W_i) = \log(\hat{\sigma}^2_{i,G}) + \frac{2(\Delta_{i,G})}{N} \quad (27)$$

where $\hat{\sigma}^2_{i,G} = \left(X_i - \left(\Phi_{i,G}\hat{\theta}_{i,G}\right)\right)^T \left(X_i - \left(\Phi_{i,G}\hat{\theta}_{i,G}\right)\right) / N$, $\Delta_{i,G} = S_i+T_i+W_i+1$

$$AIC(S_p, T_p, W_p) = \log(\hat{\sigma}^2_{p,M}) + \frac{2(\Delta_{p,M})}{N} \quad (28)$$

where $\hat{\sigma}^2_{p,M} = \left(R_p - \left(\Phi_{p,M}\hat{\theta}_{p,M}\right)\right)^T \left(R_p - \left(\Phi_{p,M}\hat{\theta}_{p,M}\right)\right) / N$, $\Delta_{p,M} = S_p+T_p+W_p+1$

$$AIC(S_q, T_q, W_q) = \log(\hat{\sigma}^2_{q,L}) + \frac{2(\Delta_{q,L})}{N} \quad (29)$$

where $\hat{\sigma}^2_{q,L} = \left(L_q - \left(\Phi_{q,L}\hat{\theta}_{q,L}\right)\right)^T \left(L_q - \left(\Phi_{q,L}\hat{\theta}_{q,L}\right)\right) / N$, $\Delta_{q,L} = S_q+T_q+W_p+1$

In (26), $\hat{\sigma}^2_{s,P}$ and $\Delta_{s,P}$ represent the estimated residual error and number (order) of parameters of the sth protein in the estimation parameter problem (22) of the PPIN, respectively; $\hat{\sigma}^2_{i,G}$ and $\Delta_{i,G}$ represent the estimated residual error and the number of parameters of the ith gene in the estimation parameter problem (23) of the GRN,

respectively; $\hat{\sigma}^2_{p,M}$ and $\Delta_{p,M}$ denote the estimated residual error and the number of parameters of the pth miRNA in the estimation parameter problem (24) of the GRN, respectively; $\hat{\sigma}^2_{q,L}$ and $\Delta_{q,L}$ represent the estimated residual error and the number of parameters of the qth lncRNA in the estimation parameter problem (25) of the GRN, respectively; $\hat{\theta}_{s,P}$ denotes the estimated parameters of the sth protein by (22); $\hat{\theta}_{i,G}$, $\hat{\theta}_{p,M}$ and $\hat{\theta}_{q,L}$ represent the estimated parameters of the ith gene by (23), the pth miRNA by (24), and the qth lncRNA by (25) in the regulatory model, respectively;

According to system identification theory of system modeling [5,6], the real system order of system model could minimize AIC. In order to obtain real system order of GENs, we minimize AIC (i.e. $AIC(S_s)$, $AIC(S_i, T_i, W_i)$, $AIC(S_p, T_p, W_p)$, and $AIC(S_q, T_q, W_q)$) of system order detection method to obtain the real number (order) of system model, i.e., S_s^* for the sth protein, S_i^*, T_i^*, W_i^* for the ith gene, S_p^*, T_p^*, W_p^* for the pth miRNA, and S_q^*, T_q^*, W_q^* for the qth lncRNA. Therefore, we could delete the insignificant interactions and regulations out of true number identified by AIC as false-positives of interactions and regulations in the candidate GENs to obtain real GENs of each stage of PTC. Finally, we obtained the real GENs of each stage PTC cell through NGS data and DNA methylation profiles as shown in Figure 2~Figure 4.

1.3 Applying the PNP method to extract core GENs in the real GENs

Real GENs are still very complex. It is still very difficult to investigate the carcinogenic progression mechanisms of different stages of PTC (i.e. from normal stage to early stage and from early stage to late stage). Therefore, we applied the PNP method on the real GRNs to extract the corresponding core GENs in each PTC stage. By the

real GENs, the system models of interactions and regulations in PPIN and GRN can be shown in the followings:

$$y_s[n] = \sum_{g\in S_s^*} \hat{\alpha}_{sg} y_g[n] y_s[n] + \hat{b}_s + v_s[n] \text{, for } s=1, \ldots, S^* \text{ and } n=1, \ldots, N^* \tag{30}$$

$$x_i[n] = \sum_{s\in S_i^*} \hat{\beta}_{is} y_s[n] M_i[n] + \sum_{t\in T_i^*} \hat{\tau}_{it} l_t[n] M_i[n] - \sum_{w\in W_i^*} \hat{\delta}_{iw} x_i[n] r_w[n] M_i[n] + \hat{k}_i M_i[n] + \varepsilon_i[n],$$

for i=1, ...,I^* and n=1, ...,N^* (31)

$$r_p[n] = \sum_{s\in S_p^*} \hat{\lambda}_{ps} y_s[n] M_p[n] + \sum_{t= T_p^*} \hat{\mu}_{pt} l_t[n] M_p[n] - \sum_{w= W_p^*} \hat{\psi}_{pw} r_p[n] r_w[n] M_p[n] + \hat{e}_p M_p[n] + \omega_p[n],$$

for p=1, ...,P^* and n=1, ...,N^* (32)

$$l_q[n] = \sum_{s\in S_q^*} \hat{\gamma}_{qs} y_s[n] M_q[n] + \sum_{t\in T_q^*} \hat{\rho}_{qt} l_t[n] M_q[n] - \sum_{w\in W_q^*} \hat{\zeta}_{qw} l_q[n] r_w[n] M_q[n] + \hat{f}_q M_q[n] + \eta_q[n],$$

for q=1, ...,Q^* and n=1, ...,N^* (33)

where $\hat{\alpha}_{sg}$ represents the estimated protein interactive abilities on the sth protein, and $\hat{\beta}_{is}$, $\hat{\lambda}_{ps}$, $\hat{\gamma}_{qs}$ denote the estimated transcriptional regulatory abilities of the sth TFs on the ith gene, the pth miRNA and the qth lncRNA, respectively; $\hat{\tau}_{it}$, $\hat{\mu}_{pt}$, $\hat{\rho}_{qt}$ reprsent the estimated transcriptional regulatory abilities of the tth lncRNAs on the ith gene, the pth miRNA and the qth lncRNA, respectively; $\hat{\delta}_{iw}$, $\hat{\psi}_{pw}$, $\hat{\zeta}_{qw}$ denote the estimated post-transcriptional regulatory abilities of the wth miRNAs on the ith gene, the pth miRNA and the qth lncRNA, respectively; S_s^*, S_i^*, T_i^*, W_i^*, S_p^*, T_p^*, W_p^*, S_q^*, T_q^* and W_q^* indicate the PPIs number of the sth protein, TF regulations on the ith gene, lncRNA regulations on the ith gene, miRNA regulations on the ith gene, TF regulations on the pth miRNA, lncRNA regulations on the pth miRNA, miRNA regulations on the

pth miRNA, TF regulations on the qth lncRNA, lncRNA regulations on the qth lncRNA, and miRNA regulations on the qth lncRNA in real GENs by AIC, respectively. Through the system identification scheme in (22), (23), (24), (25) we could estimate the above interactive and regulatory abilities. With these estimated parameters of interactive abilities of proteins, transcriptional regulatory abilities of TFs, transcriptional regulatory abilities of lncRNAs and post-transcriptional regulatory abilities of miRNAs in the real GRNs, the network matrix of real GRNs could be represented as follows:

$$Z = \begin{bmatrix} Z_{pp} & 0 & 0 \\ Z_{tg} & Z_{lg} & Z_{mg} \\ Z_{tl} & Z_{ll} & Z_{ml} \\ Z_{tm} & Z_{lm} & Z_{mm} \end{bmatrix} \in R^{\left(S^*+I^*+Q^*+P^*\right)\times\left(S^*+T^*+W^*\right)} \tag{34}$$

where the sub-network matrix $Z_{pp}=\begin{bmatrix} \hat{\alpha}_{11} & \cdots & \hat{\alpha}_{1g} & \cdots & \hat{\alpha}_{1S} \\ \vdots & \ddots & \vdots & \ddots & \vdots \\ \hat{\alpha}_{s1} & \cdots & \hat{\alpha}_{sg} & \cdots & \hat{\alpha}_{sS} \\ \vdots & \ddots & \vdots & \ddots & \vdots \\ \hat{\alpha}_{S1} & \cdots & \hat{\alpha}_{Sg} & \cdots & \hat{\alpha}_{SS} \end{bmatrix}$ represents the system matrix associated with interactive abilities of proteins; the sub-network matrices

$Z_{tg}=\begin{bmatrix} \hat{\beta}_{11} & \cdots & \hat{\beta}_{1s} & \cdots & \hat{\beta}_{1S} \\ \vdots & \ddots & \vdots & \ddots & \vdots \\ \hat{\beta}_{i1} & \cdots & \hat{\beta}_{is} & \cdots & \hat{\beta}_{iS} \\ \vdots & \ddots & \vdots & \ddots & \vdots \\ \hat{\beta}_{I1} & \cdots & \hat{\beta}_{Is} & \cdots & \hat{\beta}_{IS} \end{bmatrix}$, $Z_{lg}=\begin{bmatrix} \hat{\tau}_{11} & \cdots & \hat{\tau}_{1t} & \cdots & \hat{\tau}_{1T} \\ \vdots & \ddots & \vdots & \ddots & \vdots \\ \hat{\tau}_{i1} & \cdots & \hat{\tau}_{it} & \cdots & \hat{\tau}_{iT} \\ \vdots & \ddots & \vdots & \ddots & \vdots \\ \hat{\tau}_{I1} & \cdots & \hat{\tau}_{It} & \cdots & \hat{\tau}_{IT} \end{bmatrix}$, $Z_{mg}=\begin{bmatrix} -\hat{\delta}_{11} & \cdots & -\hat{\delta}_{1w} & \cdots & -\hat{\delta}_{1W} \\ \vdots & \ddots & \vdots & \ddots & \vdots \\ -\hat{\delta}_{i1} & \cdots & -\hat{\delta}_{iw} & \cdots & -\hat{\delta}_{iW} \\ \vdots & \ddots & \vdots & \ddots & \vdots \\ -\hat{\delta}_{I1} & \cdots & -\hat{\delta}_{Iw} & \cdots & -\hat{\delta}_{IW} \end{bmatrix}$ represent the corresponding system matrices associated with TFs transcriptional regulatory abilities, lncRNAs transcriptional regulatory abilities and miRNAs post-transcriptional regulatory abilities on genes, respectively;

The sub-network matrix $Z_{tl}=\begin{bmatrix} \hat{\gamma}_{11} & \cdots & \hat{\gamma}_{1s} & \cdots & \hat{\gamma}_{1S} \\ \vdots & \ddots & \vdots & \ddots & \vdots \\ \hat{\gamma}_{q1} & \cdots & \hat{\gamma}_{qs} & \cdots & \hat{\gamma}_{qS} \\ \vdots & \ddots & \vdots & \ddots & \vdots \\ \hat{\gamma}_{Q1} & \cdots & \hat{\gamma}_{Qs} & \cdots & \hat{\gamma}_{QS} \end{bmatrix}$, $Z_{ll}=\begin{bmatrix} \hat{\rho}_{11} & \cdots & \hat{\rho}_{1t} & \cdots & \hat{\rho}_{1T} \\ \vdots & \ddots & \vdots & \ddots & \vdots \\ \hat{\rho}_{q1} & \cdots & \hat{\rho}_{qt} & \cdots & \hat{\rho}_{qT} \\ \vdots & \ddots & \vdots & \ddots & \vdots \\ \hat{\rho}_{Q1} & \cdots & \hat{\rho}_{Qt} & \cdots & \hat{\rho}_{QT} \end{bmatrix}$, $Z_{ml}=\begin{bmatrix} -\hat{\zeta}_{11} & \cdots & -\hat{\zeta}_{1w} & \cdots & -\hat{\zeta}_{1W} \\ \vdots & \ddots & \vdots & \ddots & \vdots \\ -\hat{\zeta}_{q1} & \cdots & -\hat{\zeta}_{qw} & \cdots & -\hat{\zeta}_{qW} \\ \vdots & \ddots & \vdots & \ddots & \vdots \\ -\hat{\zeta}_{Q1} & \cdots & -\hat{\zeta}_{Qw} & \cdots & -\hat{\zeta}_{QW} \end{bmatrix}$ represent the system matrices associated with TFs transcriptional regulatory abilities, lncRNAs transcriptional regulatory abilities and miRNAs post-transcriptional regulatory abilities on lncRNAs, respectively; The sub-network matrix

$Z_{tm}=\begin{bmatrix} \hat{\lambda}_{11} & \cdots & \hat{\lambda}_{1s} & \cdots & \hat{\lambda}_{1S} \\ \vdots & \ddots & \vdots & \ddots & \vdots \\ \hat{\lambda}_{p1} & \cdots & \hat{\lambda}_{ps} & \cdots & \hat{\lambda}_{pS} \\ \vdots & \ddots & \vdots & \ddots & \vdots \\ \hat{\lambda}_{P1} & \cdots & \hat{\lambda}_{Ps} & \cdots & \hat{\lambda}_{PS} \end{bmatrix}$, $Z_{lm}=\begin{bmatrix} \hat{\mu}_{11} & \cdots & \hat{\mu}_{1t} & \cdots & \hat{\mu}_{1T} \\ \vdots & \ddots & \vdots & \ddots & \vdots \\ \hat{\mu}_{p1} & \cdots & \hat{\mu}_{pt} & \cdots & \hat{\mu}_{pT} \\ \vdots & \ddots & \vdots & \ddots & \vdots \\ \hat{\mu}_{P1} & \cdots & \hat{\mu}_{Pt} & \cdots & \hat{\mu}_{PT} \end{bmatrix}$, $Z_{mm}=\begin{bmatrix} -\hat{\psi}_{11} & \cdots & -\hat{\psi}_{1w} & \cdots & -\hat{\psi}_{1W} \\ \vdots & \ddots & \vdots & \ddots & \vdots \\ -\hat{\psi}_{p1} & \cdots & -\hat{\psi}_{pw} & \cdots & -\hat{\psi}_{pW} \\ \vdots & \ddots & \vdots & \ddots & \vdots \\ -\hat{\psi}_{P1} & \cdots & -\hat{\psi}_{Pw} & \cdots & -\hat{\psi}_{PW} \end{bmatrix}$ represent the system matrices associated with TFs transcriptional regulatory abilities, lncRNAs transcriptional regulatory abilities and miRNAs post-transcriptional regulatory abilities on miRNAs, respectively. If a protein is not interactive or regulatory with protein, gene, lncRNA and miRNA, the corresponding parameters will disappear in the pruning proceed by AIC and are padded with zero in the system network matrix H.

The Principal network projection method (PNP) was applied to the system network matrix Z to extract core GEN in real GEN of each stage PTC, and the PNP is based on

the singular value decomposition method in the following:

$$Z = EUH^{T} \tag{35}$$

where $E \in R^{\left(S^*+I^*+Q^*+P^*\right)\times\left(S^*+T^*+W^*\right)}$ and $H \in R^{\left(S^*+I^*+Q^*+P^*\right)\times\left(S^*+T^*+W^*\right)}$ are the unitary matrix; $U \in R^{\left(S^*+T^*+W^*\right)\times\left(S^*+T^*+W^*\right)}$ is diagonal matrix (i.e., $U = diag\left(u_1, \cdots, u_m, \cdots, u_{S^*+T^*+W^*}\right)$), which is composed of $S^*+T^*+W^*$ singular values of Z in a descending order (i.e., $u_1 \geq \cdots \geq u_m \geq \cdots \geq u_{S^*+T^*+W^*} \geq 0$). For example, $diag\left(u_1, u_2\right)$ denotes the diagonal matrix of u_1 and u_2 (i.e., $\begin{bmatrix} u_1 & 0 \\ 0 & u_2 \end{bmatrix}$). The eigen expression fraction (D_m) can be defined as follows:

$$D_m = \frac{u_m^2}{\sum_{m=1}^{S+T+W} u_m^2} \tag{36}$$

We choose the top M singular vectors of network matrix Z, such that $\sum_{m=1}^{M} D_m \geq 0.85$ with the minimum M to find 85% of principal network structure from the energy perspective of the real GEN i.e., top M singular vectors consist of 85% network matrix Z. Then, we show that the projection of network matrix Z to the top M singular vectors of H is given as follows:

$$H(c,r) = z_{c,:} \cdot h_{:,r}^{T}, \tag{37}$$

for $c = 1,\ldots,S+I+Q+P$ and $r = 1,\ldots,M$

where $z_{c,:}$ denotes the cth row vector of Z; $h_{:,r}$ denotes the rth row vector of H. We therefore define the 2-norm projection value of each node of proteins, genes, miRNAs and lncRNAs in the real GEN to the top M right singular vectors in the following:

$$B(c) = [\sum_{r=1}^{M} H^2(c,r)]^{1/2}, \qquad (38)$$

for $c = 1,\ldots,S+I+Q+P$ and $r = 1,\ldots,M$

where $B(c)$ is the 2-norm projection value of the cth node of core network to the 85% principal network structure of GEN. Based on the projection values $B(c)$, for example, if $B(c)$ is close to zero, the corresponding node is insignificant and nearly independent to the top 85% network structure of core network. Conversely, if the projection value of a corresponding node is higher, the node plays a more important role in the GEN principal network structure. The core GENs of PTC in normal stage, early stage and late stage through PNP method are shown Figure 3, Figure 4 and Figure 5, respectively.

We projected these core GENs to KEGG pathways to construct the corresponding core signaling pathways to investigate significant molecular mechanisms of carcinogenic progression in the consecutive stages of PTC (i.e. from normal to early stage and from early stage to late stage). From carcinogenic progression mechanisms, we could identify significant biomarkers as drug targets. Further, from the changes of the core protein basal level between two consecutive stages more than a threshold, we could predict the core protein to interact with acetyltransferase proteins, deacetylase proteins, ubiquitin proteins and deubiquitinase proteins directly or not depending on whether the interacting epigenetic enzyme expression level is higher than the standard threshold or not. Finally, if the change of a core gene basal level between two consecutive stages is higher than a threshold, then it may mainly influence by the DNA methylation.

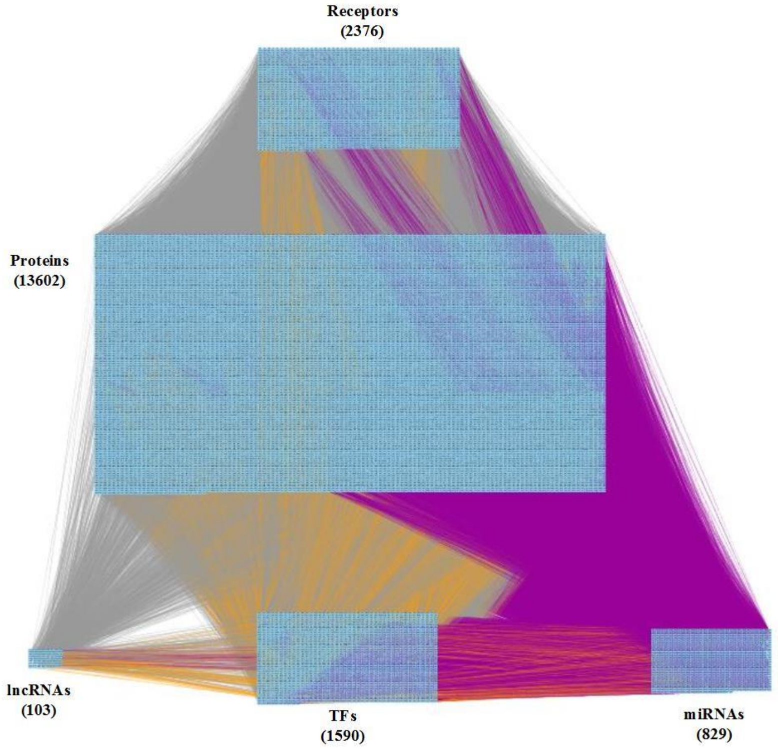

Figure S1. The genetic and epigenetic network (GEN) of normal stage of thyroid cells.

The figure represents the identified GENs of normal thyroid cells. The grey lines indicate protein-protein interactions (PPIs); The orange lines denote transcriptional regulations by TFs and lncRNAs; The purple lines represent post-transcriptional regulations by miRNAs; The numbers of Receptors, Proteins, lncRNAs, TFs and miRNAs are 2376, 13602, 103, 1590 and 829, respectively.

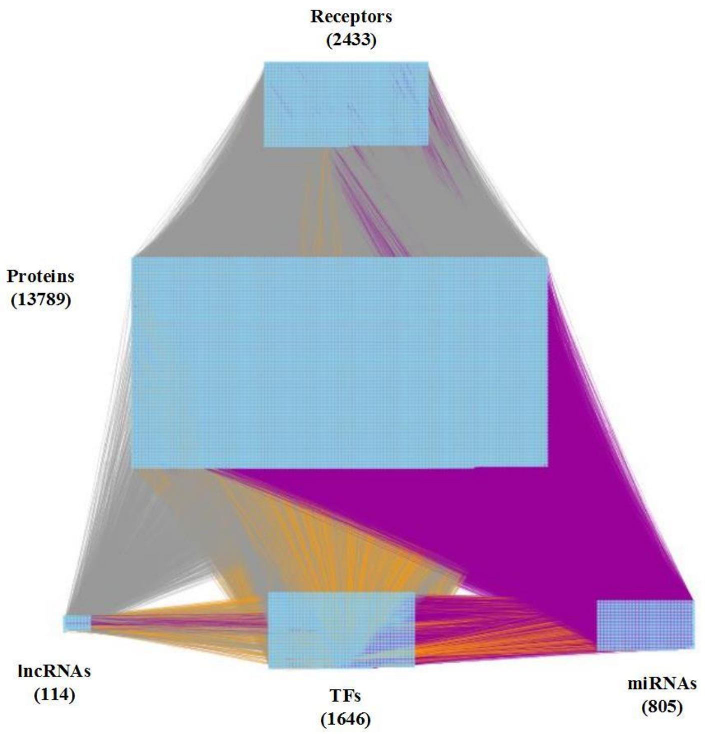

Figure S2. The genetic and epigenetic network (GEN) of early-stage of thyroid cancer cells.

The figure represents the identified GENs of early-stage thyroid cells. The grey lines indicate protein-protein interactions (PPIs); The orange lines denote transcriptional regulations by TFs and lncRNAs; The purple lines represent post-transcriptional regulations by miRNAs; The numbers of Receptors, Proteins, lncRNAs, TFs and miRNAs are 2433, 13789, 114, 1646 and 805, respectively.

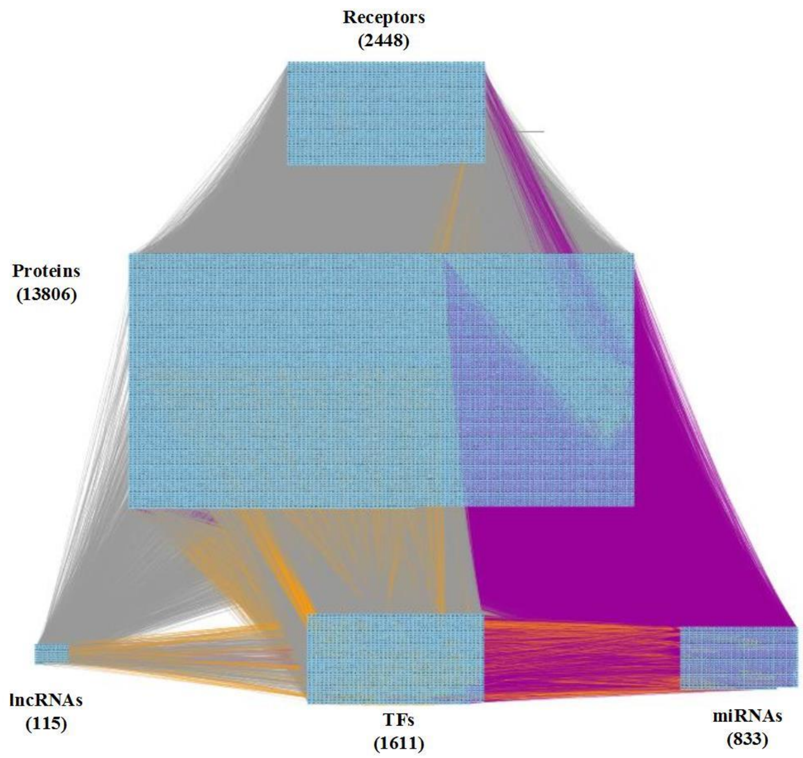

Figure S3. The genetic and epigenetic network (GEN) of late-stage of thyroid cancer cells.

The figure represents the identified GENs of late-stage thyroid cells. The grey lines indicate protein-protein interactions (PPIs); The orange lines denote transcriptional regulations by TFs and lncRNAs; The purple lines represent post-transcriptional regulations by miRNAs; The numbers of Receptors, Proteins, lncRNAs, TFs and miRNAs are 2448, 13806, 115, 1611 and 833, respectively.

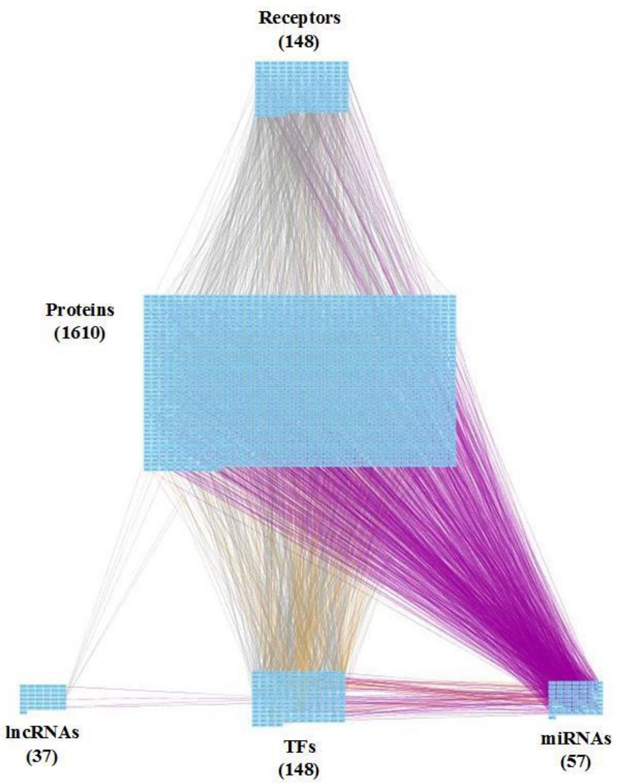

Figure S4. The core genetic and epigenetic network (GEN) of normal stage of thyroid cells. The figure represents the identified core GEN of normal thyroid cells. The grey lines indicate protein-protein interactions (PPIs); The orange lines denote transcriptional regulations by TFs and lncRNAs; The purple lines represent post-transcriptional regulations by miRNAs; The numbers of Receptors, Proteins, lncRNAs, TFs and miRNAs are 148, 1610, 37, 148 and 57, respectively.

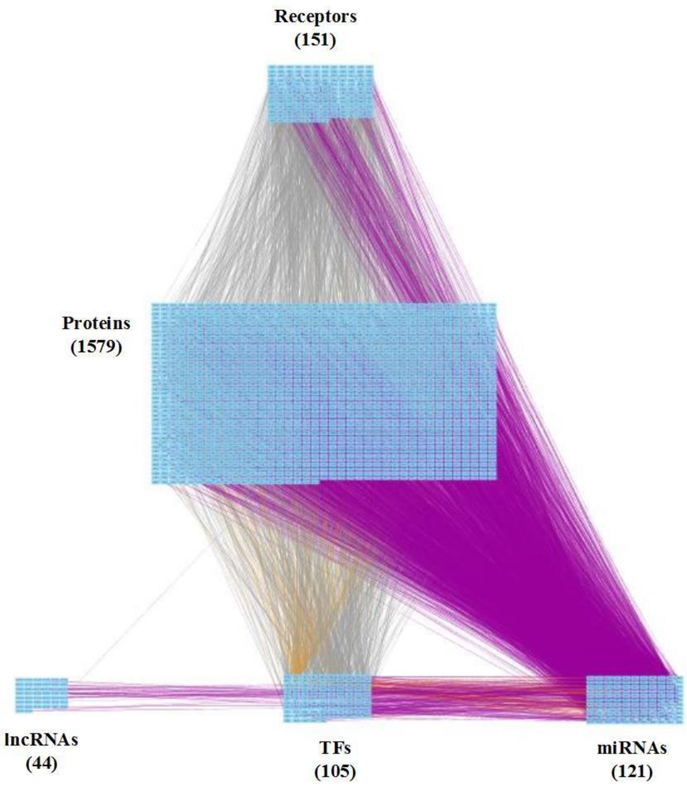

Figure S5. The core genetic and epigenetic network (GEN) of early-stage of thyroid cancer cells. The figure represents the identified core GEN of early-stage thyroid cells. The grey lines indicate protein-protein interactions (PPIs); The orange lines denote transcriptional regulations by TFs and lncRNAs; The purple lines represent post-transcriptional regulations by miRNAs; The numbers of Receptors, Proteins, lncRNAs, TFs and miRNAs are 151, 1579, 44, 105 and 121, respectively.

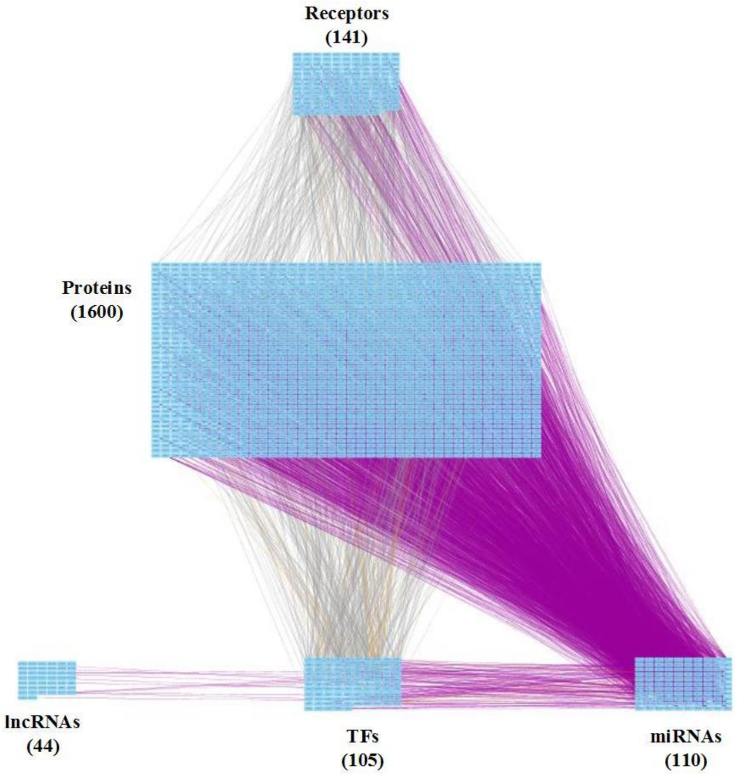

Figure S6. The core genetic and epigenetic network (GEN) of late-stage of thyroid cancer cells. The figure represents the identified core GEN of late-stage thyroid cells. The grey lines indicate protein-protein interactions (PPIs); The orange lines denote transcriptional regulations by TFs and lncRNAs; The purple lines represent post-transcriptional regulations by miRNAs; The numbers of Receptors, Proteins, lncRNAs, TFs and miRNAs are 141, 1600, 44, 105 and 110, respectively.

Reference

1. Bandres, E.; Agirre, X.; Bitarte, N.; Ramirez, N.; Zarate, R.; Roman-Gomez, J.; Prosper, F.; Garcia-Foncillas, J. Epigenetic regulation of microrna expression in colorectal cancer. *Int J Cancer* **2009**, *125*, 2737-2743.

2. Medvedeva, Y.A.; Khamis, A.M.; Kulakovskiy, I.V.; Ba-Alawi, W.; Bhuyan, M.S.I.; Kawaji, H.; Lassmann, T.; Harbers, M.; Forrest, A.R.R.; Bajic, V.B. Effects of cytosine methylation on transcription factor binding sites. *BMC Genomics* **2014**, *15*, 119-119.

3. Weber, M.; Hellmann, I.; Stadler, M.B.; Ramos, L.; Paabo, S.; Rebhan, M.; Schubeler, D. Distribution, silencing potential and evolutionary impact of promoter DNA methylation in the human genome. *Nat Genet* **2007**, *39*, 457-466.

4. Li, C.W.; Chen, B.S. Network biomarkers of bladder cancer based on a genome-wide genetic and epigenetic network derived from next-generation sequencing data. *Dis Markers* **2016**, *2016*, 4149608.

5. Chen, B.S.; Wu, C.C. Systems biology as an integrated platform for bioinformatics, systems synthetic biology, and systems metabolic engineering. *Cells* **2013**, *2*, 635-688.

6. Chen, B.S.; Li, C.W. Big mechanisms in systems biology-big data mining, network modeling, and genome-wide data identification. *Elsevier* **2017**, 878.
